# Identification of SARS-CoV-2 Nucleocapsid and Spike T-Cell Epitopes for Assessing T-Cell Immunity

**DOI:** 10.1128/JVI.02002-20

**Published:** 2021-02-24

**Authors:** Eunok Lee, Kerrie Sandgren, Gabriel Duette, Vicki V. Stylianou, Rajiv Khanna, John-Sebastian Eden, Emily Blyth, David Gottlieb, Anthony L. Cunningham, Sarah Palmer

**Affiliations:** a Centre for Virus Research, The Westmead Institute for Medical Research, Westmead, New South Wales, Australia; b Sydney Medical School, Faculty of Medicine and Health, The University of Sydney, Camperdown, New South Wales, Australia; c Faculty of Medicine, The University of Queensland, Brisbane, Queensland, Australia; d QIMR Berghofer Centre for Immunotherapy and Vaccine Development, QIMR Berghofer Medical Research Institute, Brisbane, Queensland, Australia; e Centre for Infectious Diseases and Microbiology, The Westmead Institute for Medical Research, Westmead, New South Wales, Australia; f Marie Bashir Institute for Infectious Diseases and Biosecurity, School of Life and Environmental Sciences and School of Medical Sciences, The University of Sydney, Westmead, New South Wales, Australia; g Centre for Cancer Research, The Westmead Institute for Medical Research, Westmead, New South Wales, Australia; h BMT and Cell Therapies Program, Westmead Hospital, Westmead, New South Wales, Australia; The Peter Doherty Institute for Infection and Immunity

**Keywords:** COVID-19 vaccines, diagnostic tools, nucleocapsid and spike proteins, protein network, SARS-CoV-2, T-cell effector/polyfunctionality, highly networked/conserved T-cell epitope derived peptides

## Abstract

Developing optimal T-cell response assays to severe acute respiratory syndrome coronavirus type 2 (SARS-CoV-2) is critical for measuring the duration of immunity to this disease and assessing the efficacy of vaccine candidates. These assays need to target conserved regions of SARS-CoV-2 global variants and avoid cross-reactivity to seasonal human coronaviruses. To contribute to this effort, we employed an *in silico* immunoinformatics analysis pipeline to identify immunogenic peptides resulting from conserved and highly networked regions with topological importance from the SARS-CoV-2 nucleocapsid and spike proteins. A total of 57 highly networked T-cell epitopes that are conserved across geographic viral variants were identified from these viral proteins, with a binding potential to diverse HLA alleles and 80 to 100% global population coverage. Importantly, 18 of these T-cell epitope derived peptides had limited homology to seasonal human coronaviruses making them promising candidates for SARS-CoV-2-specific T-cell immunity assays. Moreover, two of the NC-derived peptides elicited effector/polyfunctional responses of CD8^+^ T cells derived from SARS-CoV-2 convalescent patients.

**IMPORTANCE** The development of specific and validated immunologic tools is critical for understanding the level and duration of the cellular response induced by SARS-CoV-2 infection and/or vaccines against this novel coronavirus disease. To contribute to this effort, we employed an immunoinformatics analysis pipeline to define 57 SARS-CoV-2 immunogenic peptides within topologically important regions of the nucleocapsid (NC) and spike (S) proteins that will be effective for detecting cellular immune responses in 80 to 100% of the global population. Our immunoinformatics analysis revealed that 18 of these peptides had limited homology to circulating seasonal human coronaviruses and therefore are promising candidates for distinguishing SARS-CoV-2-specific immune responses from pre-existing coronavirus immunity. Importantly, CD8^+^ T cells derived from SARS-CoV-2 survivors exhibited polyfunctional effector responses to two novel NC-derived peptides identified as HLA-binders. These studies provide a proof of concept that our immunoinformatics analysis pipeline identifies novel immunogens which can elicit polyfunctional SARS-CoV-2-specific T-cell responses.

## INTRODUCTION

As of October 2020, there are over 36 million known cases of COVID-19 worldwide, which is caused by infection with severe acute respiratory syndrome coronavirus 2 (SARS-CoV-2). In order to combat this pandemic, vaccines are rapidly being developed to reduce the risk and spread of this infection (1–6). Recently, a clinical trial involving a vaccine that contains the spike glycoprotein (S) of SARS-CoV-2 showed T-cell responses were elicited at day 14 and antibodies against the virus at day 28 post-vaccination for the majority of participants (7). However, the longitudinal assessment of the level and duration of both T-cell immunity and antibodies elicited by this and other SARS-CoV-2 vaccines is required.

Previously, it has been shown that antibody levels wane with time in SARS-CoV-1 infection, while cellular immunity can last 6 to 11 years (8–13). Similarly, a recent study of antibody levels revealed that 40% of asymptomatic and 13% of symptomatic patients infected by SARS-CoV-2 became negative for immunoglobulin G eight weeks post-recovery (14). Exposing T cells from recovered SARS-CoV-1 patients to peptides derived from the S protein of this virus revealed that the induction of polyfunctional T cells (T cells producing multiple effector cytokines) was higher in individuals with severe SARS-CoV-1 infection than in those with moderate infection, indicating that the level of T-cell response corresponds with the severity of this infection and time to recovery (15). A recent study in recovered COVID-19 patients revealed that even in the absence of antibodies to SARS-CoV-2, a robust T-cell immune response was measured, indicating the importance of T-cell immunity in response to COVID-19 (16). In particular, T-cell activation/exhaustion and lymphopenia were associated with severe disease, whereas traditional effector functions of CD8^+^ T cells were related to a better prognosis (17). Since the cellular responses during COVID-19 are complex, longitudinal assessment of both CD4^+^ and CD8^+^ T-cell responses can inform how SARS-CoV-2 infection and vaccines for this disease modulate immune functions over time.

DNA vaccines containing the S gene derived from SARS-CoV-1 and Middle East Respiratory Syndrome (MERS)-CoV have been shown to induce T-cell responses in mice and humans, respectively (18–20). In addition, SARS-CoV-1 nucleocapsid (NC) protein has been shown to stimulate strong T-cell responses in monkeys and mice (21–25). Similarly, specific NC-derived peptides have been shown to induce cellular response from both CD4^+^ and CD8^+^ T-cell subsets derived from patients recovered from SARS-CoV-1 and SARS-CoV-2 (16, 26–29). All current SARS-CoV-2 vaccines include the S protein and a robust T-cell immunity against NC-derived peptides can be detected in convalescent COVID-19 patients (1–6, 16, 26, 29). Therefore, identifying T-cell epitope derived peptides within these two viral proteins will provide effective tools for measuring T-cell responses in COVID-19 patients with different degrees of disease severity and/or evaluating immunogenicity of vaccine candidates in clinical trials.

There are several challenges when developing immunogen peptides for the assessment of SARS-CoV-2-specific T-cell immunity that can be applied globally. First, the genetic profile of SARS-CoV-2 can be region specific, complicating the detection of the T-cell immunity against global viral variants (30, 31). Second, SARS-CoV-2-reactive CD4^+^ T cells were identified in 40 to 60% of unexposed individuals, suggesting cross-reactive T-cell recognition between the four circulating human coronaviruses (229E, HKU1, NL63, and OC43) which cause the common cold and SARS-CoV-2 (32–34). Third, human leukocyte antigen (HLA) alleles are extremely polymorphic with more than 18,000 HLA class I and 7,000 class II alleles currently reported (35). Keeping these three points in mind, in order to measure SARS-CoV-2-specific T-cell immune responses, it will be important to select SARS-CoV-2 peptide antigens for T-cell response tests that reflect all global viral variants and are not cross-reactive with other human coronaviruses, rather than using complete libraries of overlapping peptides. In addition, selecting peptide antigens that can bind to diverse HLA alleles will be critical.

Importantly, recent research has highlighted the significance of regions within a viral protein comprised of “highly networked” amino acids (36). These regions have topological importance to tertiary and quaternary viral protein structure and are not frequently mutated. In human immunodeficiency virus (HIV)-infected individuals with diverse HLA class I alleles, targeting epitopes from these highly networked regions with cytotoxic T cells provided virological control. Therefore, determining immunogenic peptides derived from highly networked regions of the SARS-CoV-2 proteins will be a priority to ensure coverage of all emerging strains of SARS-CoV-2. In addition, a combination of these immunogenic peptides will be superior to using the whole viral spike protein when measuring T-cell responses in patients recovered from COVID-19 since they will increase the sensitivity of the assay to worldwide strains and will distinguish SARS-CoV-2-specific responses from cross-reactive seasonal coronavirus responses. Moreover, the combination of these highly networked T-cell epitope derived peptides has the potential to bind to diverse HLA alleles.

In the present study, we applied an immunoinformatics analysis pipeline to define immunodominant epitopes in currently circulating SARS-CoV-2 viral variants. These epitopes are restricted to HLA class I and II molecules and selected from topologically important regions of the NC and S proteins with the goal of identifying immunogenic peptides that can contribute to the development of assays for SARS-CoV-2-specific T-cell immunity in patients with different disease severity and after vaccination.

## RESULTS

### HLA class I restricted T-cell epitopes derived from SARS-CoV-2 nucleocapsid and spike proteins.

**FIG 1:**
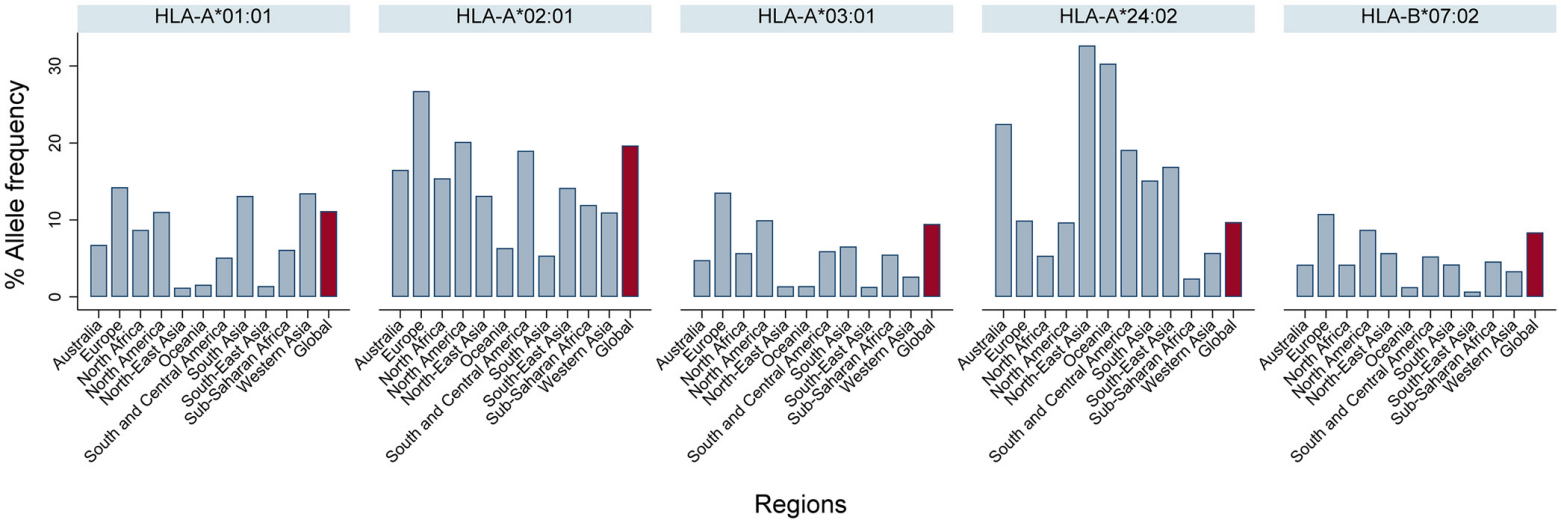
Global and regional HLA class I allele frequency. The data were curated from The Allele Frequency Net Database (www.allelefrequencies.net).

**FIG 2:**
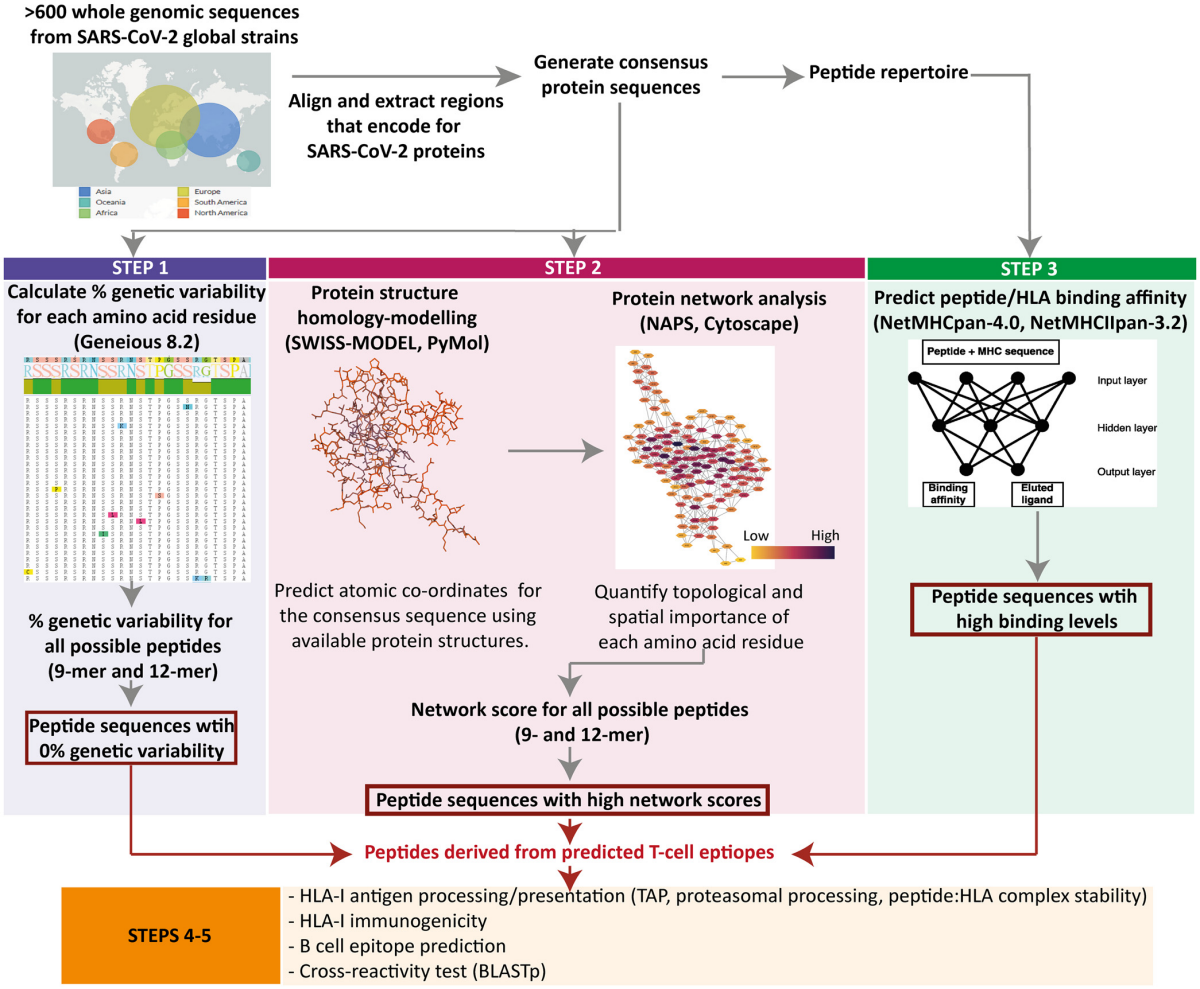
Immunoinformatics analysis pipeline. Immunoinformatics analysis pipeline used to identify highly networked SARS-CoV-2 T-cell epitope derived peptides for HLA class I- and II-restricted immune responses.

We defined 9-mer T-cell epitopes restricted to the HLA-A*02:01 allele which are conserved across geographic SARS-CoV-2 variants. HLA-A*02:01 was selected due to its worldwide prevalence (Fig. 1) (37). These epitopes matched >95% of circulating SARS-CoV-2 variants globally (The Global Initiative on Sharing All Influenza Data [GISAID], as of August 2020) (38–40). Employing our immunoinformatic analysis pipeline, we then identified those epitopes from genetically conserved regions which were comprised of amino acid residues with topological and spatial importance within the NC and S proteins of SARS-CoV-2 (Fig. 2).

**FIG 3:**
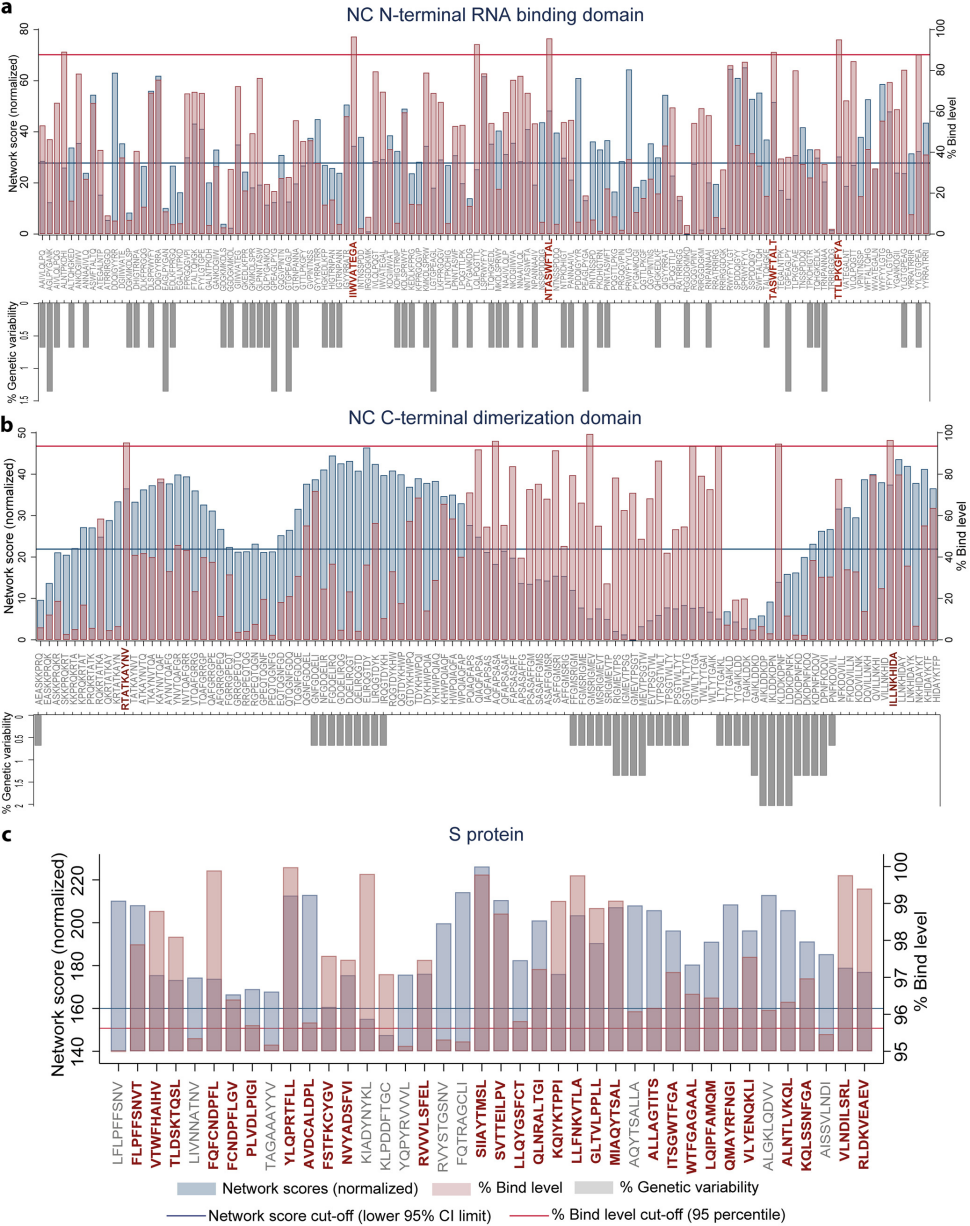
Genetically conserved 9-mer peptides with highly networked amino acid residues and restricted to HLA-A*02:01 allele. Network scores (blue bars), predicted percent bind level to the HLA class I molecule (pink bars) and percent genetic variability across SARS-CoV-2 variants (gray bars) within the peptide repertoires derived from SARS-CoV-2 NC N-terminal RNA binding domain (a), the NC C-terminal dimerization domain (b), and the S protein (c) are shown. For the S protein, the 9-mers with the network score of at least 100 and the bind level of at least 95% are shown among the repertoire of 1112 peptides. All the network scores presented in this figure are normalized. The thresholds for network scores (blue line) are determined by the lower 95% confidence of the mean. The cutoffs for the percent bind level (red line) equates to the 95th percentile. The peptides with the network scores and percent bind level above the cutoffs and those which are conserved (i.e., 0% genetic variability) across the SARS-CoV-2 isolates are highlighted in red.

**FIG 4:**
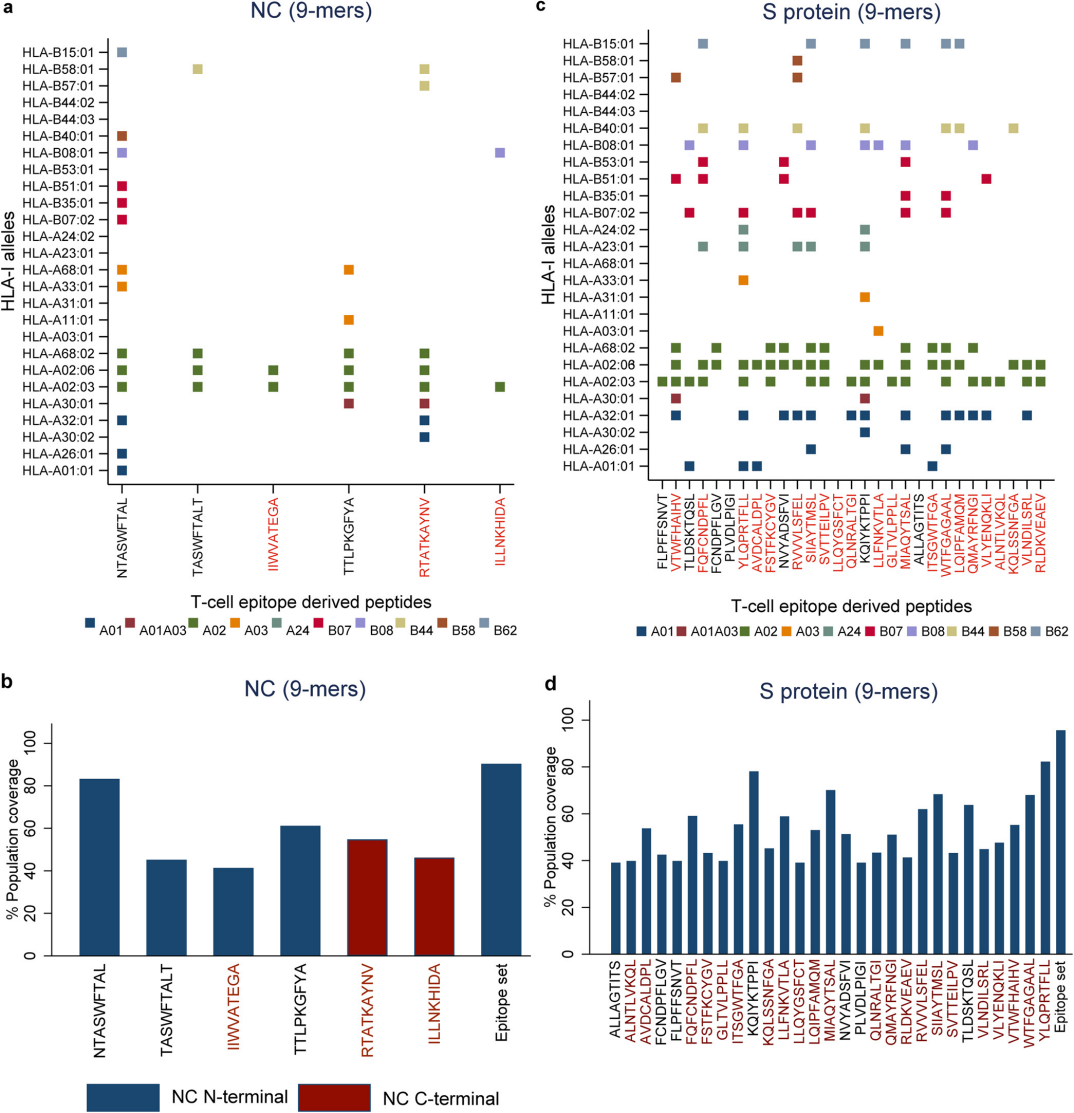
Binding prediction of highly networked T-cell epitope derived peptides to additional HLA class I alleles and global population coverage. (a and c) Binding predictions of highly networked T-cell epitope derived peptides identified from the SARS-CoV-2 NC (a) and S protein (c). The binding predictions for 9-mer T-cell epitope derived peptides to HLA-A and HLA-B alleles are classified into 10 supertypes (a and c). The peptides with top 5% bind levels to each of these additional HLA class I alleles are indicated as squares. The supertypes are indicated by different colors, as shown in the figure. (b and d) Percent global population coverage of highly networked T-cell epitope derived peptides identified from the NC (b) and S protein (d). The most promising T-cell epitope derived peptides for HLA class I-mediated immune recognition are highlighted in red.

**FIG 5:**
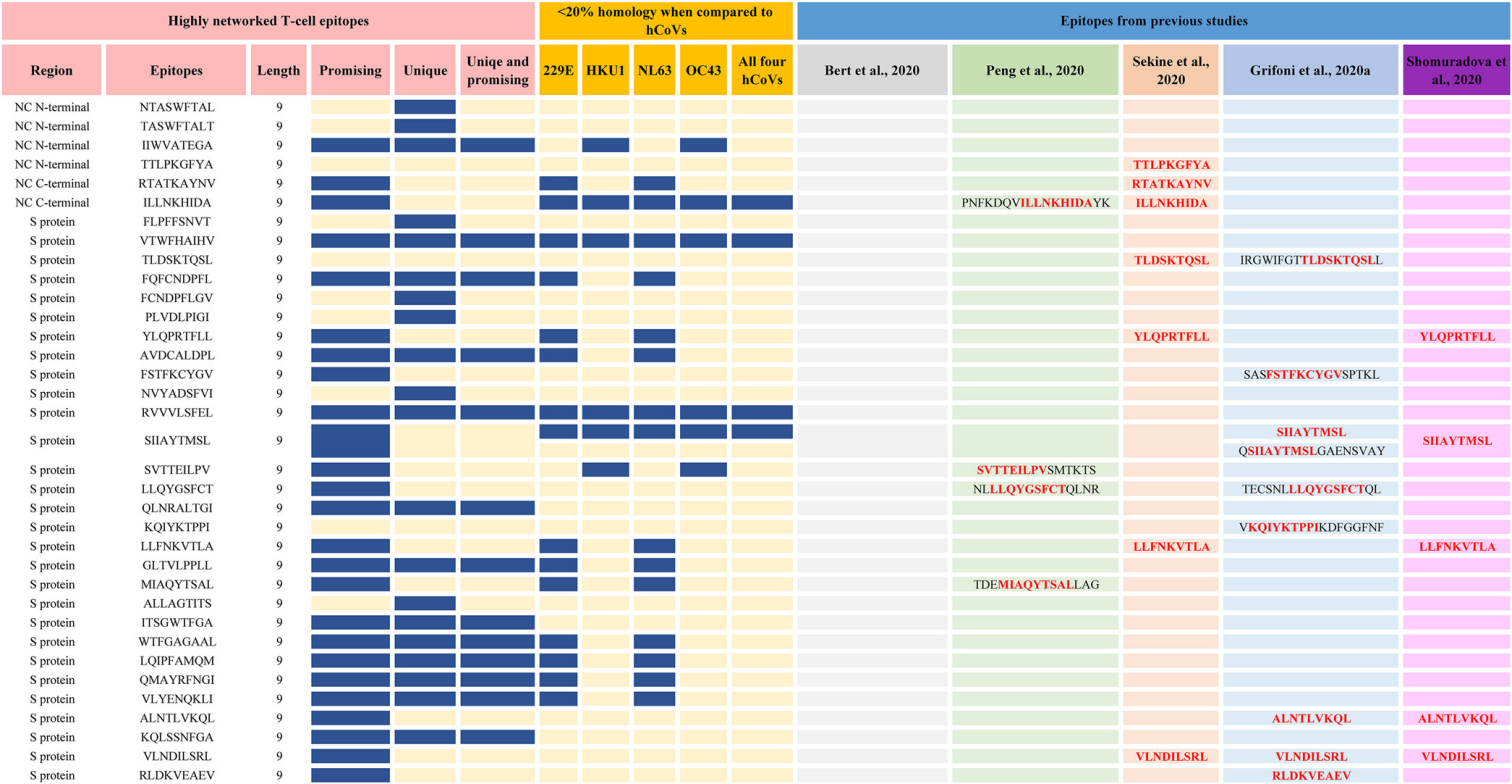
Sequence comparison between highly networked T-cell epitope derived peptides identified by immunoinformatics analysis pipeline and other studies (9-mers). The highly networked T-cell epitope derived peptides (9-mers) were compared to the epitopes identified by Le Bert et al. (26) (gray), Grifoni et al. (43) (sky blue), Shomuradova et al. (44) (pink), Sekine et al. (16) (orange), and Peng et al. (29) (green). The highly networked T-cell epitopes which are most promising (i.e., those with top 5% scores for HLA class I-mediated antigen processing and immunogenicity parameters), unique, or both are indicated by blue rectangles in the figure. Also, the most promising peptides for HLA class I-restricted immune response with <20% homology to four seasonal coronaviruses are indicated by blue rectangles.

We computed the network and spatial importance of the T-cell epitopes within N-terminal RNA binding and C-terminal dimerization domains modeled from the NC consensus protein sequence (Fig. 2) (41, 42). We identified a total of six T-cell epitope derived peptides (9-mer) from the highly networked and conserved regions of the NC protein (four from the N-terminal domain and two from the C-terminal domain; Table 1 and Fig. 3a and b). Of note, together these six peptides can bind to multiple HLA class I alleles which cover approximately 90% of the global population (Fig. 4a and b). We also compared all six peptides from the NC protein with high network scores and percent bind levels (i.e., a high binding capacity to HLAs) to the SARS-CoV-2 epitopes identified within recent publications and the Immune Epitope Database and Analysis Resource (IEDB) (Table 1 and Fig. 5) (16, 26, 29, 43, 44). We found three unique T-cell epitope derived peptides which did not contain the full complement of consecutive amino acid residues identified by these recent studies and the IEDB (16, 26, 29, 43, 44).

**TABLE 1:**
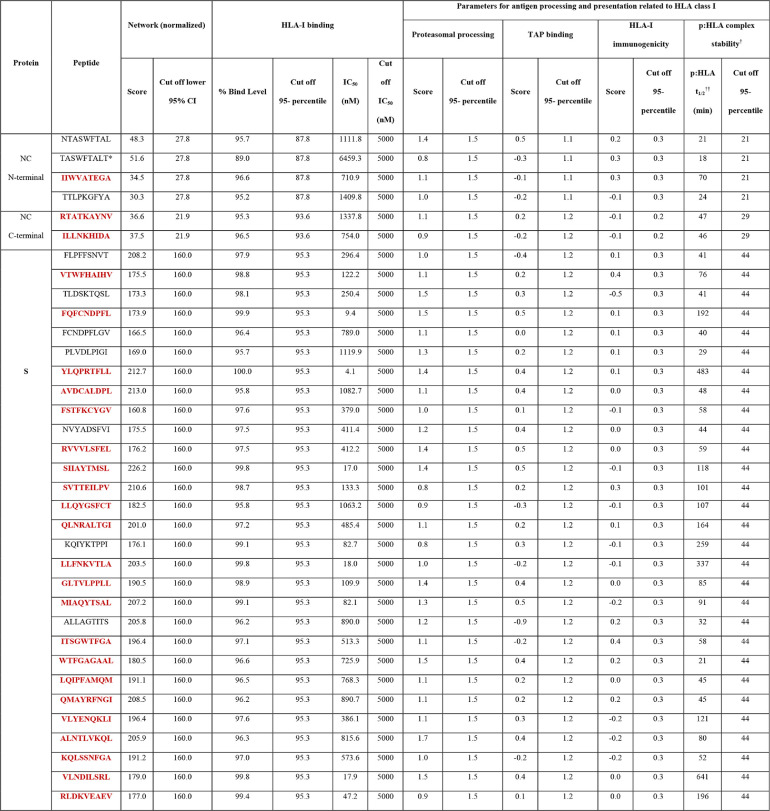
Highly networked 9-mer peptides with binding potential to HLA class I molecule (HLA-A*02:01)^*a*^

a Highlighted in red are the most promising T-cell epitope derived peptides for HLA class I-mediated immune recognition (i.e., those with top 5% scores for HLA class I antigen processing and presentation parameters). *, This peptide has a high network score and a percent bind level above the 95th percentile; however, the IC_50_ is above the cutoff IC_50_ for HLA-I binding. †, p:HLA, peptide and HLA-A*02:01 complex; ††, p:HLA *t*_1/2_, predicted time required for the dissociation of peptide:HLA complex.

For the N-terminal domain, we identified a total of four T-cell epitope derived peptides that had high network scores above the acceptable threshold (Table 1 and Fig. 3a). This indicates that these T-cell epitope derived peptides are found within the core areas of the tertiary structure of the N-terminal domain. Also, these peptides identified from the N-terminal domain had a binding capacity to the HLA-A*02:01 molecule above the 95th-percentile cutoff (bind levels, 95.2 to 96.6%; Table 1). Moreover, three of these peptides had strong to moderate binding affinities to HLA class I (half-maximal inhibitory concentration [IC_50_]) of 711 to 1,410 nM (Table 1). However, only one of these T-cell epitope derived peptides (IIWVATEGA) was considered a promising immunogenic peptide since it reached our threshold of the top 5% for the HLA class I antigen processing and presentation parameters (i.e., immunogenicity prediction and peptide:HLA complex stability; Table 1). Of note, this peptide selected by our immunoinformatics algorithm is unique and has not been identified by recent studies (Fig. 5) (16, 26, 29, 43, 44).

For the C-terminal domain, we identified two T-cell epitope derived peptides (ILLNKHIDA and RTATKAYNV) that comprised of highly networked amino acid residues and high binding levels to the HLA class I molecule (Table 1 and Fig. 3b). Also, these peptides reached our threshold of the top 5% for the predicted HLA class I antigen processing and presentation parameters (i.e., the stability of the peptide:HLA complex; Table 1), making them the most promising candidates for T-cell immunity assays from the C-terminal domain. Taken together, three T-cell epitopes (IIWVATEGA, RTATKAYNV, and ILLNKHIDA) among the peptide repertoire derived from the NC protein had the most promising properties for HLA class I-restricted antigen presentation. For these three highly networked T-cell epitope derived peptides selected from the NC protein, we then assessed whether they could bind to additional HLA class I alleles other than HLA-A*02:01 (Fig. 4a). Our analysis predicted that these peptides had binding capacity for two to six additional HLA-A and HLA-B alleles which are classified into different HLA class I supertypes (45). The peptides individually can cover approximately 41 to 55% of the global population (Fig. 4b).

**FIG 6:**
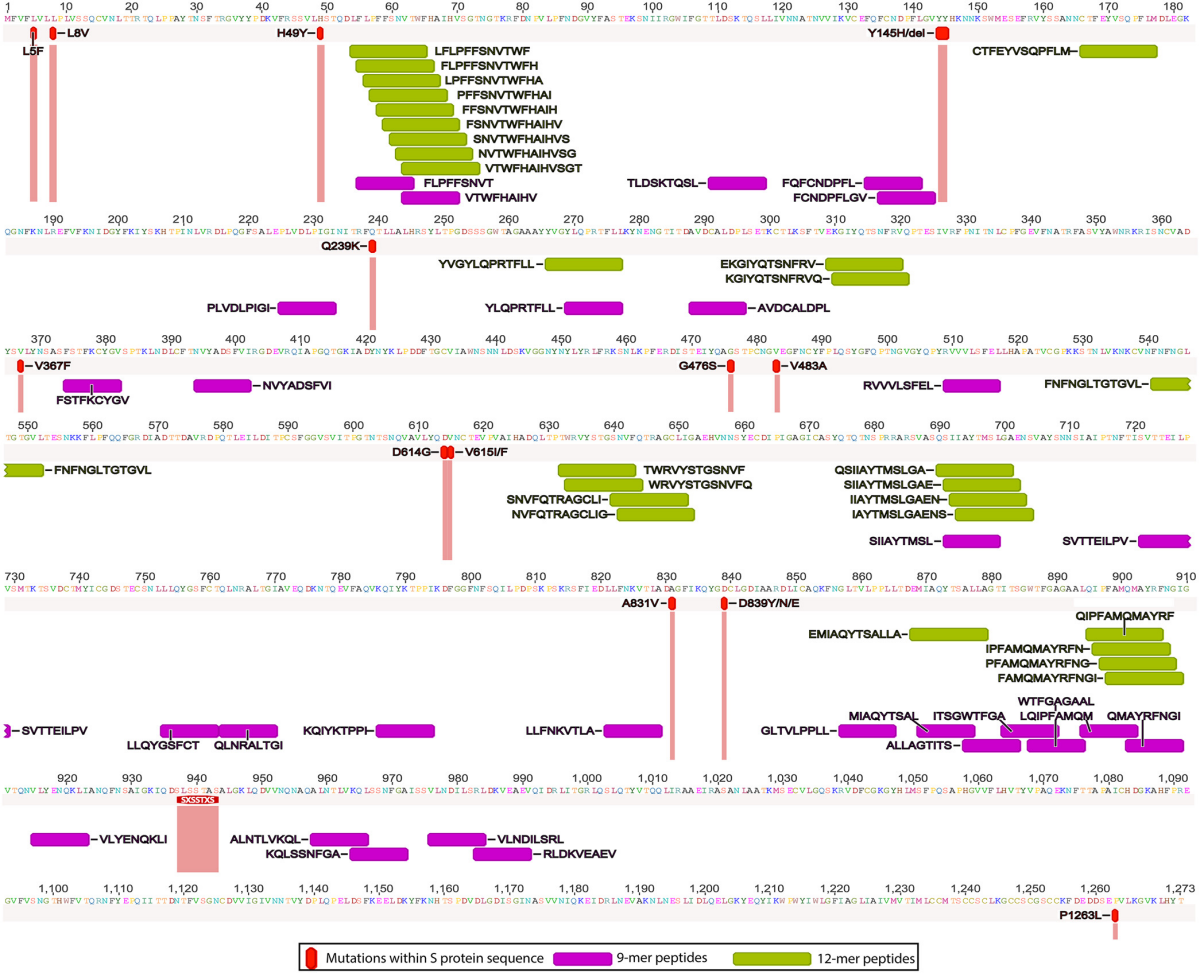
Comparison between highly networked T-cell epitope derived peptides identified from SARS-CoV-2 S protein and mutation sites that enhance viral infectivity. The highly networked T-cell epitope derived peptides are mapped to the consensus S protein sequence with 14 mutation sites that enhance viral infectivity. The mutation sites have been identified by Korber et al. (30).

For the S protein, we found only 2.6% of the T-cell epitope derived peptides contain highly networked amino acid residues with a binding potential to the HLA class I molecule (29 of 1,112 9-mer peptides; Table 1 and Fig. 3c). Importantly, none of these peptides were derived from the regions within the S protein which contain mutations reported to enhance viral infectivity (Fig. 6) (30). In addition, the peptides had the potential to bind to 23 additional HLA class I alleles which are classified into 10 supertypes (Fig. 4c) (45). These 29 peptides selected from the topologically important regions of the S protein can cover up to 96% of the global population when combined (Fig. 4d). A total of 12 of 29 T-cell epitope derived peptides selected by our immunoinformatics analysis pipeline contained consecutive amino acid residues which were 100% identical to the epitopes identified by other groups (Fig. 5) (16, 26, 29, 43, 44).

Of these 29 highly networked T-cell epitope derived peptides determined from the S protein, we identified 22 peptides which reached the top 5% for the HLA class I antigen processing and presentation parameters (Table 1). This indicates that the protein regions comprised of these epitopes are the most promising sites for T-cell immune recognition. However, we found that 10 of these 22 T-cell epitopes have been previously described by other groups (Table 1 and Fig. 5) (16, 26, 29, 43, 44). This immunoinformatics pipeline allowed us to identify 12 unique T-cell epitopes within the S protein as promising for HLA class I restricted immune response.

### Highly networked T-cell epitopes derived from the SARS-CoV-2 nucleocapsid are correlated with HLA class I antigen processing and presentation parameters.

**FIG 7:**
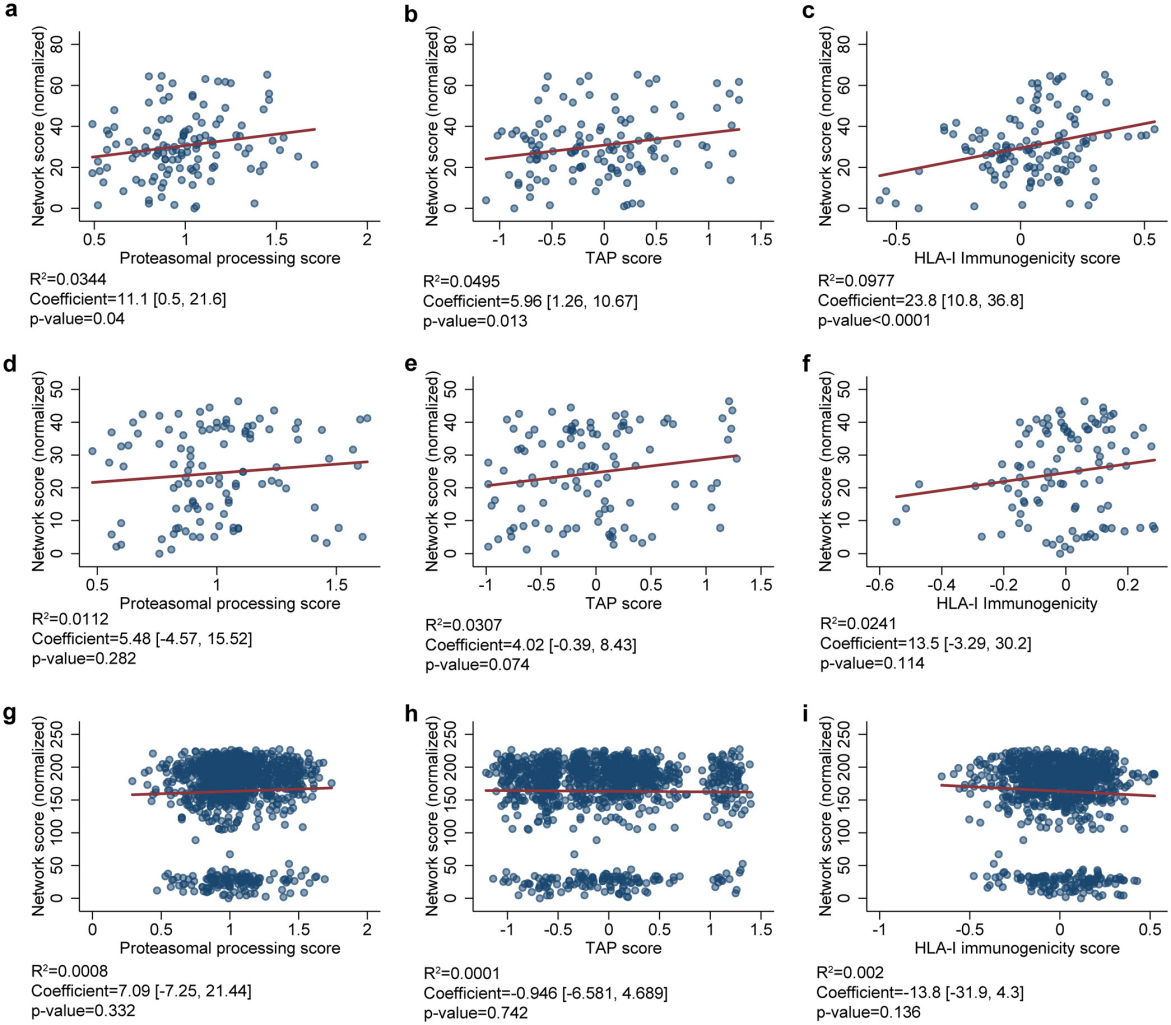
Correlation between network scores and predictions for HLA class I-mediated antigen processing and presentation. The correlations between the normalized network scores and the predictions for proteasomal processing (a, d, and g), TAP binding (b, e, and h), and HLA class I-mediated immunogenicity (c, f, and i) are shown. The data includes 123 peptides derived from the NC N-terminal domain (a to c), 110 peptides derived from the NC C-terminal domain (d to f), and 1,112 peptides from the S protein (g to i). The *R*^2^ value indicates the degree of the peptide repertoire that follow linear regression and the coefficient indicates the slope of the regression. The 95% confidence intervals (in square brackets), and the *P* value for the coefficient are also presented.

To compare whether the structural topology of T-cell epitopes correlates to the HLA class I-related antigen processing and presentation, we determined the association between the highly networked T-cell epitope derived peptides within the SARS-CoV-2 tertiary structures and the HLA class I-mediated immune restriction (Fig. 7). We found the network scores for the T-cell epitope derived peptides from the NC, particularly the N-terminal domain, were positively correlated with HLA class I antigen processing and presentation parameters (*P* < 0.0001 to 0.282; Fig. 7a to f). In contrast, the highly networked T-cell epitope derived peptides from the S protein were not associated with these parameters (*P* = 0.136 to 0.742; Fig. 7g to i). However, our immunoinformatics analysis pipeline identified a subset of the 9-mer T-cell epitope derived peptides from the S protein which were promising for HLA class I-restricted immune response (*n* = 22, Table 1).

### HLA class II-restricted T-cell epitopes derived from SARS-CoV-2 nucleocapsid and spike proteins.

**FIG 8:**
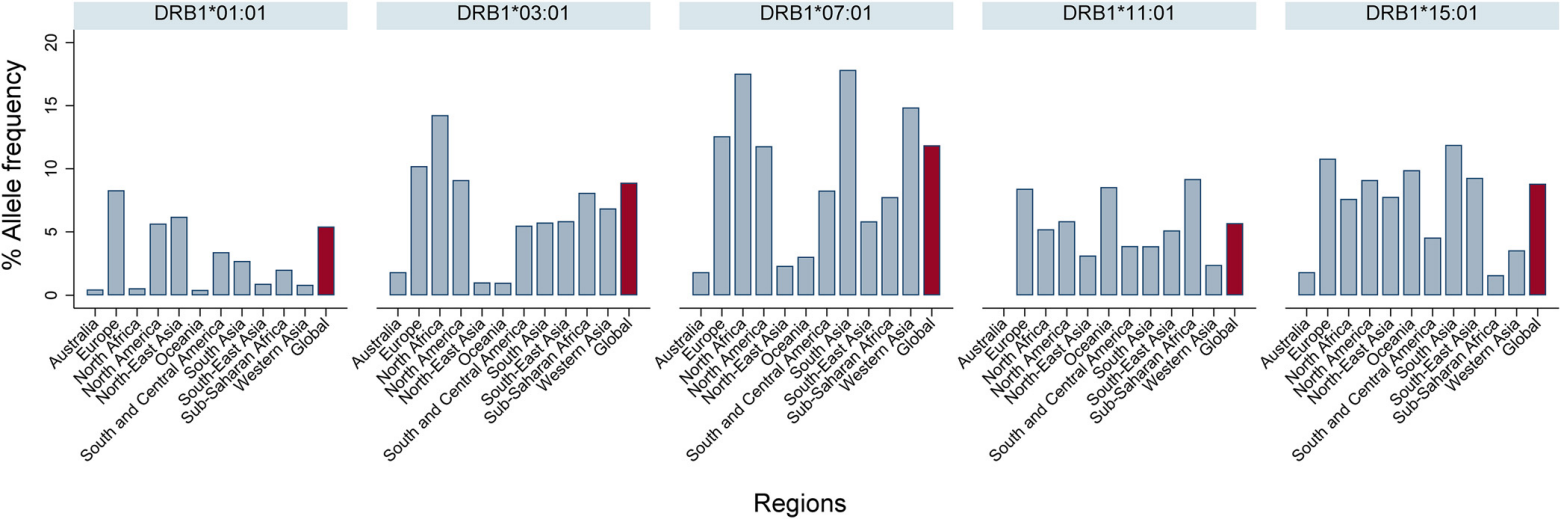
Global and regional HLA class II allele frequency. The data were curated from The Allele Frequency Net Database (www.allelefrequencies.net).

To define T-cell epitopes restricted to DRB1*07:01, HLA class II allele, we used the same immunoinformatics analysis pipeline mentioned above incorporating HLA class II binding prediction (36, 46, 47). This HLA class II allele was selected due its worldwide prevalence (Fig. 8) (37). The HLA class II molecules frequently accommodate peptides between 13 and 17 amino acids in length (48, 49). However, we focused on the peptide repertoire composed of 12 amino acid residues as this particular length is substantially associated with high binding affinity to HLA class II molecules (50). Of note, the T-cell epitope derived peptides that we identified match >95% of circulating SARS-CoV-2 variants globally (GISAID as of August 2020) (38–40).

**FIG 9:**
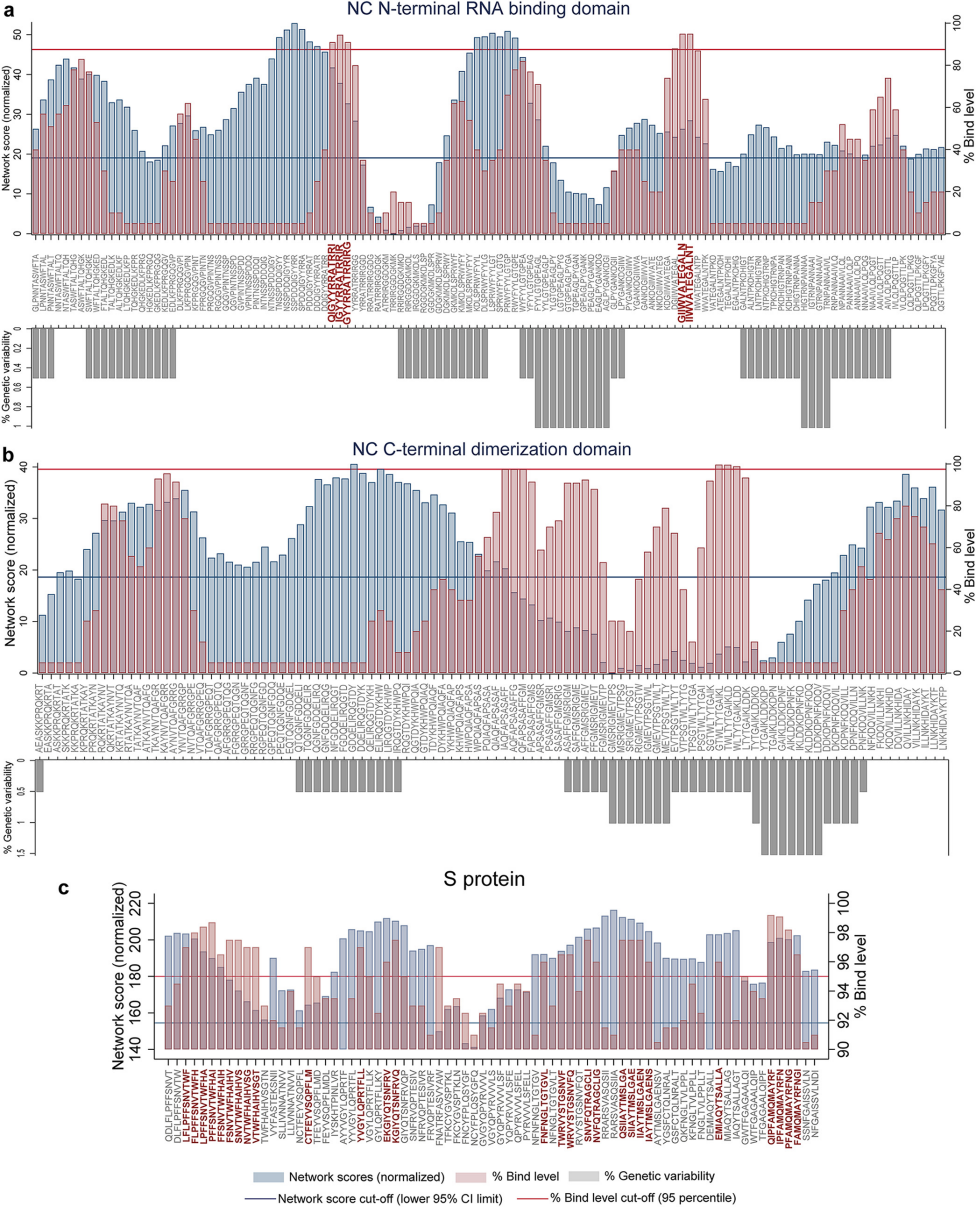
Genetically conserved 12-mer peptides with highly networked amino acid residues and restricted to DRB1*07:01 allele. Network scores (blue bars), the predicted percent bind level to the HLA class II molecule (pink bars), and the percent genetic variability across SARS-CoV-2 variants (gray bars) within the peptide repertoires derived from the SARS-CoV-2 NC N-terminal RNA binding domain (a), the NC C-terminal dimerization domain (b), and the S protein (c) are shown. For the S protein, the 12-mers with the network score of at least 100, and a bind level of at least 95% are shown among the repertoire of 1,112 peptides. All the network scores presented in this figure are normalized. The thresholds for network scores (blue line) are determined by the lower 95% confidence of the mean. The cutoffs for the percent bind level (red line) equates to the 95th percentile. The peptides with the network scores and percent bind level above the cutoffs and those which are conserved (i.e., 0% genetic variability) across the SARS-CoV-2 isolates are highlighted in red.

**FIG 10:**
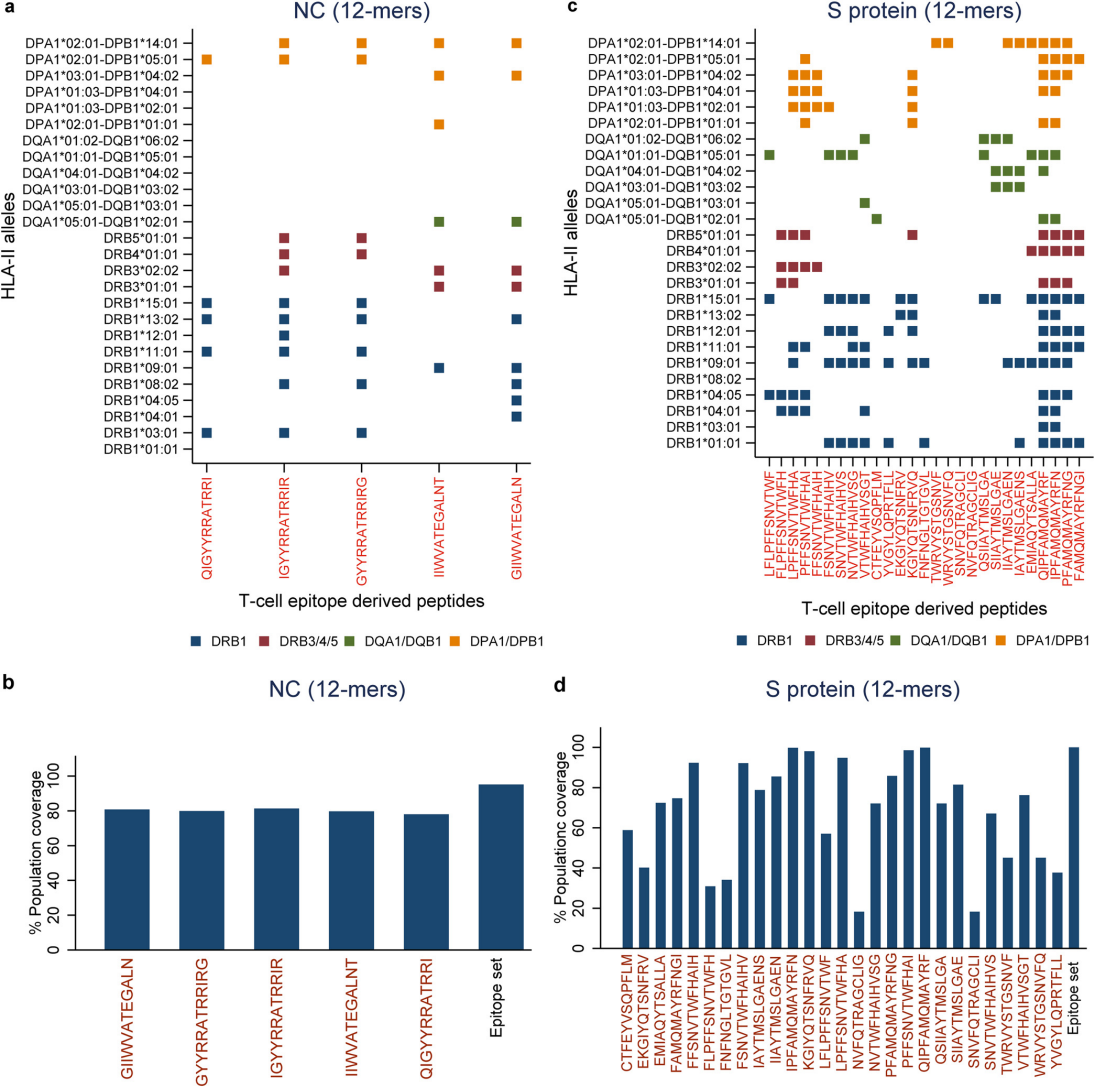
Binding prediction of highly networked T-cell epitope derived peptides to additional HLA class II alleles and global population coverage. (a and c) The binding prediction of highly networked T-cell epitope derived peptides identified from the SARS-CoV-2 NC (a) and S protein (c). The binding prediction of 12-mer T-cell epitope derived peptides to HLA class II alleles are classified into four loci. The peptides with the top 5% bind levels to each of these additional HLA class II alleles are indicated as squares. The loci are indicated by different colors, as shown in the figure. (b and d) Percent global population coverage of highly networked T-cell epitope derived peptides identified from the NC (b) and the S protein (d). The most promising T-cell epitope derived peptides for HLA class II-mediated immune recognition are highlighted in red.

**FIG 11:**
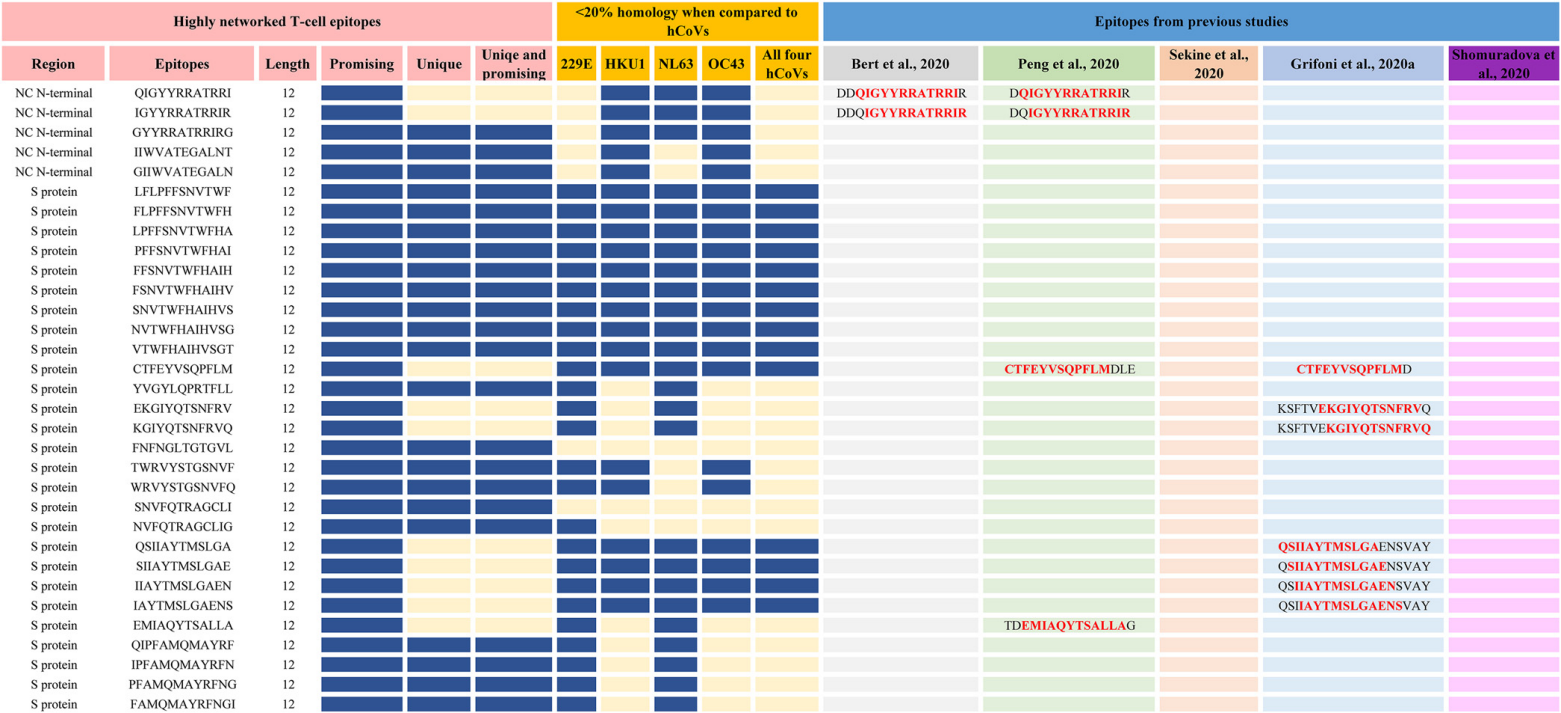
Sequence comparison between highly networked T-cell epitope derived peptides identified by immunoinformatics analysis pipeline and other studies (12-mers). The highly networked T-cell epitope derived peptides (12-mers) were compared to the epitopes identified by Le Bert et al. (26) (gray), Grifoni et al. (43) (sky blue), Shomuradova et al. (44) (pink), Sekine et al. (16) (orange), and Peng et al. (29) (green). The highly networked T-cell epitopes which are most promising (i.e., those with network scores and percent bind level above the thresholds), unique, or both are indicated by blue rectangles in the figure. Also, the most promising peptides for an HLA class II-restricted immune response with <20% homology to four seasonal coronaviruses are indicated by blue rectangles.

From a repertoire of 120 peptides derived from T-cell epitopes of the N-terminal domain of NC, we found five promising peptides which contained highly networked amino acid residues (network score range of 26.4 to 41.7, threshold of 24.6; Table 2 and Fig. 9a). These five peptides had high binding potentials to the HLA class II molecule (bind levels of 91 to 95%; and IC_50_ of 188 to 325 nM). In addition, these peptides scored within the top 5% bind level to 18 additional HLA class II alleles classified into two to four HLA-II loci (Fig. 10a) (51). Individually, these five peptides are predicted to cover approximately 80% of the global population (Fig. 10b). When all of the five peptides were combined, a global population coverage of 95% was predicted. Of these peptides derived from the highly networked regions of the NC N-terminal domain, three were found within the same B cell epitope (Table 2). Of note, only two of the five peptides derived from the NC N-terminal domain contained the full complement of consecutive amino acid residues identified by other studies (Fig. 11) (16, 26, 29, 43, 44). In contrast, none of the peptides derived from the C-terminal domain of the NC scored above the thresholds for both the protein network score and the percent bind level (Fig. 9b).

**TABLE 2:**
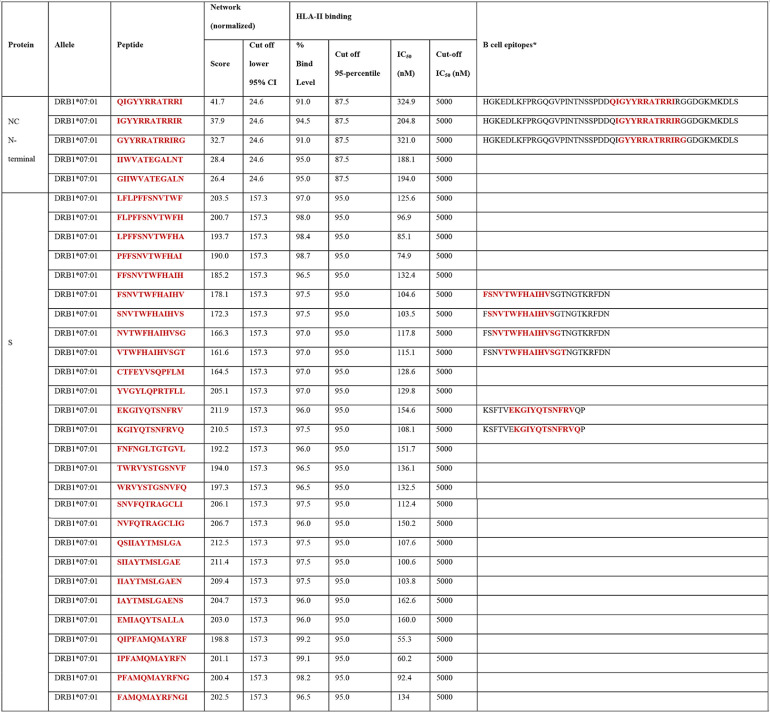
Highly networked 12-mer peptides with binding potential to HLA class II molecule (DRB1*07:01)^*a*^

a Highlighted in red are most promising T-cell epitope derived peptides for HLA class II-mediated immune recognition (i.e., those with network scores and percent bind level above the thresholds). *, B cell epitopes predicted by BepiPred Linear Epitope prediction 2.0. The consecutive amino acid residues within the B cell epitopes that are identical to the T-cell epitope derived peptides (highlighted in red).

Of 1,109 peptides derived from the S protein, we found 27 HLA class II-restricted T-cell epitope derived peptides with high network scores (Table 2 and Fig. 9c). These peptides had percent bind levels above the cutoff, as well as predicted IC_50_ values of 55.3 to 162.6 nM, making these peptides promising candidates for T-cell immunity assays. Importantly, none of these peptides were derived from the regions within the S protein which contain mutations that have the potential to enhance viral infectivity (Fig. 6) (30). Also, we found that these peptides can bind to 24 additional HLA class II alleles classified into four loci (Fig. 10c) (51). When all peptides are combined, approximately 100% global population coverage is predicted (Fig. 10d). Of these peptides, six were found in two B cell epitopes (Table 2). Moreover, we found a total of eight which were 100% identical to the epitopes identified by other studies (Table 2 and Fig. 11) (16, 26, 29, 43, 44). This immunoinformatics analysis pipeline allowed us to identify 19 new T-cell epitopes within the S protein as promising immunogenic peptides for the assays that detect T-cell immunity against SARS-CoV-2.

### Identification of highly networked T-cell epitope derived peptides within SARS-CoV-2 NC and S proteins with low homology to seasonal human coronaviruses.

To identify SARS-CoV-2 peptide antigens that can be used to assess COVID-19-specific T-cell responses and differentiate from cross-reactive seasonal coronavirus immunity, we compared the highly networked SARS-CoV-2 T-cell epitope derived peptides to four seasonal human coronaviruses (229E, HKU1, NL63, and OC43) (32–34).

**FIG 12:**
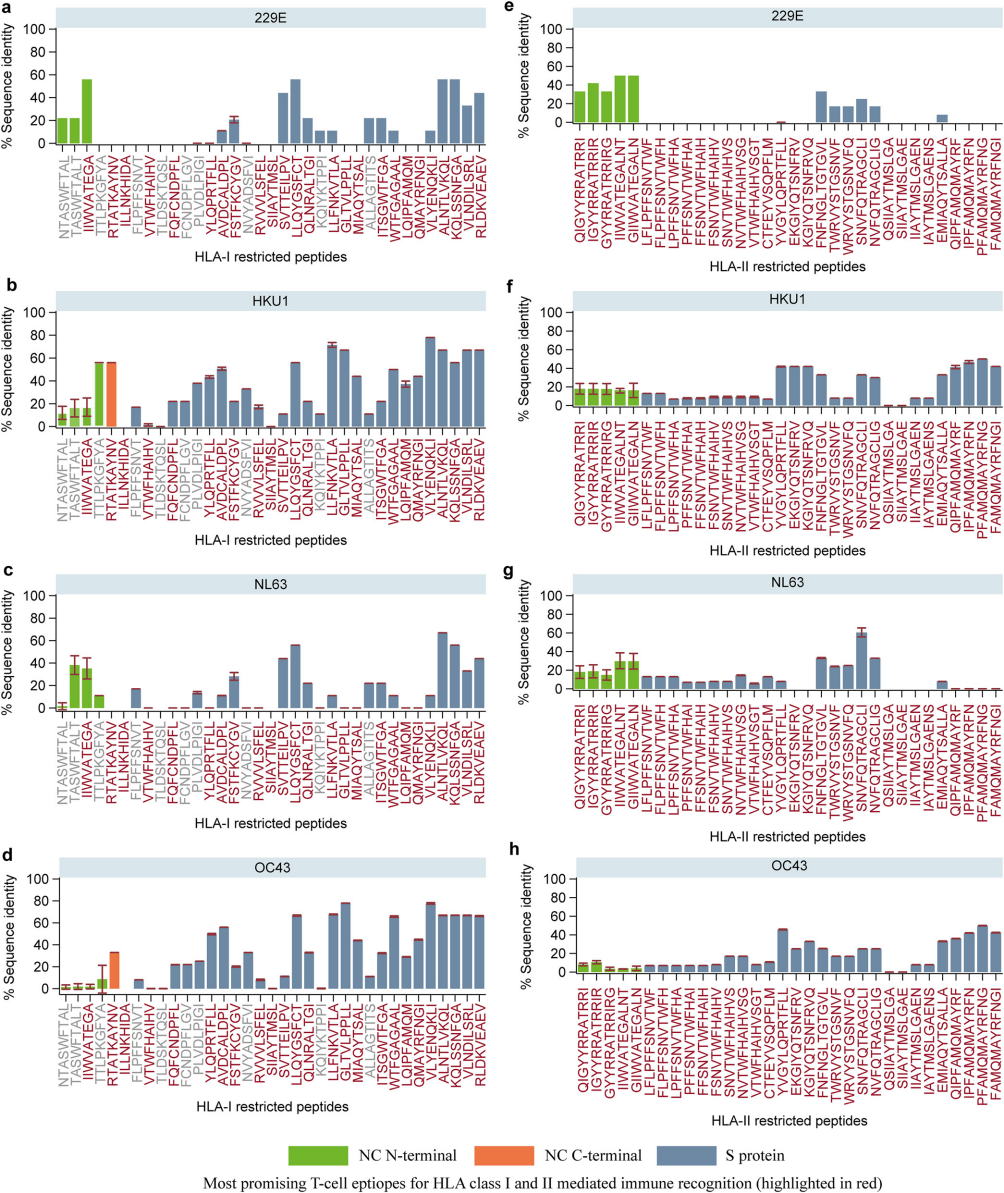
Sequence homology of highly networked T-cell epitope derived peptides identified from SARS-CoV-2 NC and S proteins to seasonal human coronaviruses. The homology between the T-cell epitope derived peptides (a to d, 9-mers; e to h, 12-mers) and four seasonal human coronaviruses (229E, HKU1, NL63, and OC43) is presented as the mean sequence identity (%) and the corresponding 95% confidence intervals. The most promising T-cell epitope derived peptides for HLA class I- and II-mediated immune recognition are highlighted in red.

For the highly networked T-cell epitope derived peptides identified within the NC protein, the most promising peptides for HLA class I (9-mers, *n* = 3) and HLA class II (12-mers, *n* = 5) had 0 to 56% homology to four human coronaviruses (Fig. 12). Of the three peptides restricted to HLA class I immune response, ILLNKHIDA had no homology to any of the seasonal human coronaviruses (Fig. 12a to d). In addition, all of the highly promising peptides restricted to HLA class II immune response had homology of <20% compared to two seasonal coronaviruses (HKU1 and OC43; Fig. 12e to h).

The highly networked T-cell epitope derived peptides identified from the S protein that were most promising for HLA class I (9-mers, *n* = 22) and class II (12-mers, *n* = 27) had 0% to 78% homology to the four seasonal coronaviruses (Fig. 12). Of note, three of the 9-mer peptides (SIIAYTMSL, VTWFHAIHV, and VTWFHAIHV) and 14 of the 12-mer peptides showed low homology to all four human coronaviruses (Fig. 12). Furthermore, a majority of 9- and 12-mer peptides had <20% homology compared to seasonal coronaviruses 229E and NL63.

Although the degree of homology between the highly networked T-cell epitope derived peptides and the four seasonal human coronaviruses is different, our immunoinformatics analysis pipeline allowed us to identify 18 peptides which were highly specific to SARS-CoV-2 (Fig. 5 and 11). Of these, we identified 11 T-cell epitope derived peptides that were unique and highly promising for HLA class I- and II-restricted immune responses that are SARS-CoV-2 specific (16, 26, 29, 43, 44).

### Highly networked T-cell epitope derived peptides bind to the HLA-A*02 molecules as predicted.

To assess whether our binding prediction is accurate for the highly networked T-cell epitope derived peptides, we selected the two most promising T-cell epitope derived peptides from the NC protein based on their HLA-I binding. In addition, these peptides were selected based on their predicted scores for the HLA class I antigen processing and presentation parameters. The peptide from the N-terminal region was selected for the *in vitro* binding validation as it had the highest bind level and the longest predicted time required for the peptide-HLA complex to dissociate (IIWVATEGA; Table 1). Also, this T-cell epitope derived peptide has not been identified by recent studies (Fig. 5) (16, 26, 29, 43, 44). The peptide from the C-terminal region was chosen since it scored the highest values for three of the four HLA class I antigen processing and presentation parameters (RTATKAYNV; Table 1). The ability of these peptides to bind to HLA-A*02:01 *in vitro* was assessed using a human-derived TAP-deficient T2 cell line (T2 cells) expressing HLA-A*02:01 on the cell surface (52, 53).

**FIG 13:**
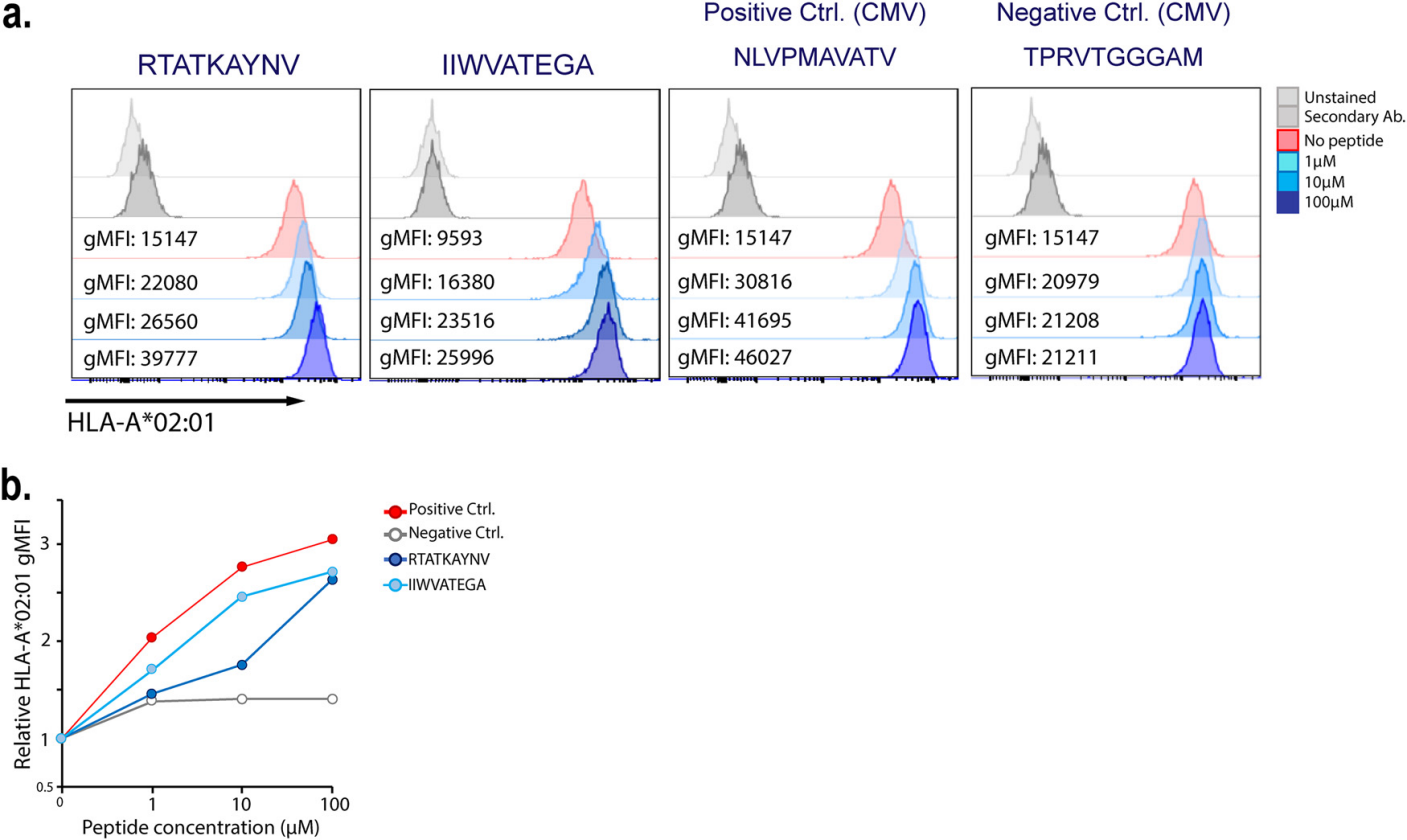
*In vitro* validation of SARS-CoV-2 NC peptides binding to HLA-A*02:01 by using T2 cells. T-cell epitope derived peptides identified from the N- and C-terminal domains of the SARS-CoV-2 NC protein were used to assess the binding capacity (RTATKAYNV and IIWVATEGA). HLA-A*02:01 expression on the cell surface was measured as gMFI by flow cytometry. NLVPMAVATV (HLA-A*02 binding) and TPRVTGGGAM (HLA-A*02 nonbinding) peptides from CMV pp65 were used as positive and negative controls. (a) Histograms from a representative experiment. (b) HLA-A*02:01 expression expressed relative to the no-peptide control. (*n* = 2).

These highly networked NC-derived peptides were able to bind and stabilize the HLA-A*02 molecules on the surface of the T2 cells (Fig. 13). The mean fluorescence intensity of the peptide:HLA-A*02 detection exceeded the non-HLA-A*02 binding control (i.e., negative control) across a series of peptide concentrations. In particular, the binding of the peptide from the NC N-terminal domain to these HLA-A*02 molecules (IIWVATEGA) was similar to the positive control EBV peptide across all concentrations (Fig. 13b).

### Effector and polyfunctional response of CD8^+^ T cells to highly networked T-cell epitope derived peptides.

**FIG 14:**
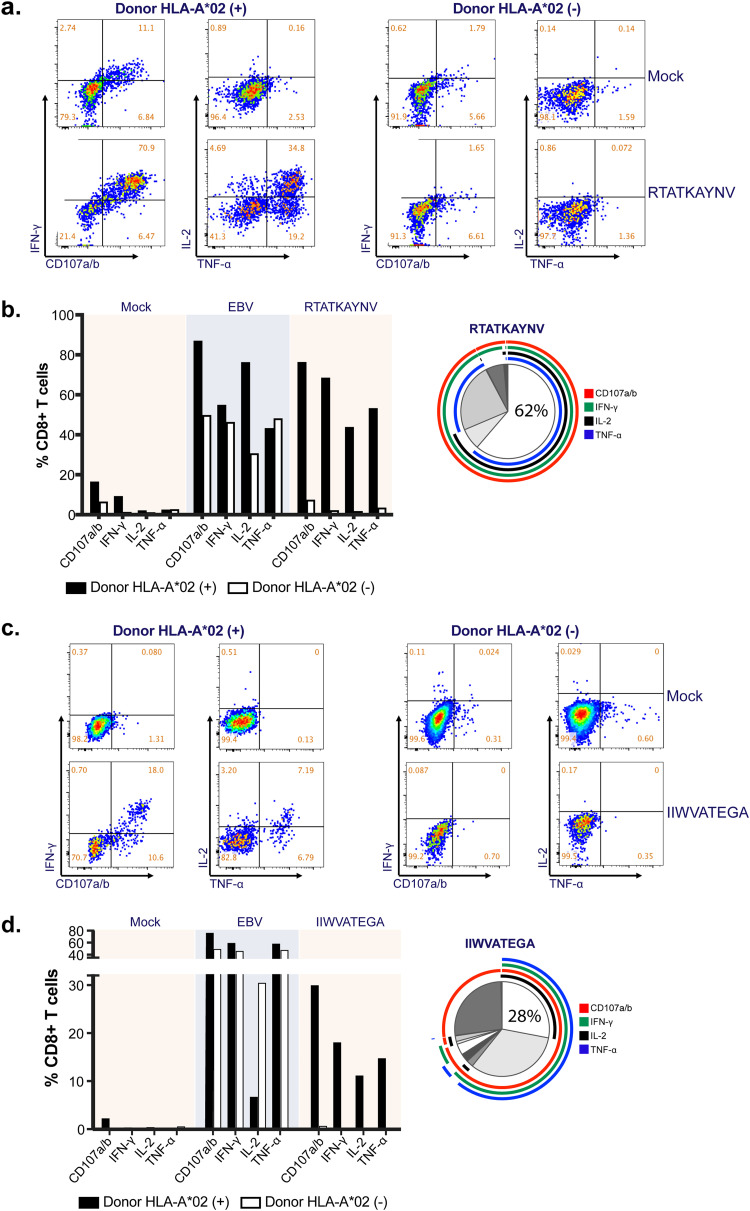
CD8^+^ T-cell polyfunctionality analysis after *ex vivo* expansion with specific SARS CoV-2 peptides derived from the SARS-CoV-2 NC. (a and c) Representative dot plots showing effector cytokine production and degranulation level of CD8^+^ T cells stimulated with RTATKAYNV (a) and IIWVATEGA (c) peptides. The CD8^+^ T cells were derived from two HLA-A*02-positive SARS-CoV-2 convalescent donors. PBMCs from an HLA-A*02-negative SARS-CoV-2 convalescent donor were used as a negative control. The clinical samples were obtained at 1 to 2 months after SARS-CoV-2 recovery. (b and d) Bar charts represent the effector profiles of RTATKAYNV (b)- and IIWVATEGA (d)-specific CD8^+^ T cells. PBMCs expanded with EBV peptides were used as positive control. Pies depict the distribution of mono-, bi-, tri-, and tetrafunctional cells within specific CD8^+^ T cells.

There is growing evidence for the importance of CD8^+^ T cells in control of SARS-CoV-2 disease severity (17). Therefore, we focused on testing the two HLA-A*02 restricted T-cell epitope derived peptides. The immunogenicity of these highly networked peptides selected from the NC protein (IIWVATEGA and RTATKAYNV) was tested in peripheral blood mononuclear cells (PBMCs) obtained from participants 1 to 2 months after recovery from SARS-CoV-2 infection (Table 3). When the PBMCs from two convalescent participants who were HLA-A*02 positive were stimulated by the peptides, we observed a robust production of interleukin-2 (IL-2), interferon gamma (IFN-γ), tumor necrosis factor-alpha (TNF-α) and a marker for the degranulation of CD8^+^ T cells (CD107a/b) (Fig. 14). This effector response was specific for HLA-A*02 (HLA-A*02:01 and HLA-A*02:06) as no such effector response was observed in the HLA-A*02-negative donor (HLA-A*24:02) (Table 3, Fig. 4a, and Fig. 14a and c). Of note, 28 to 62% of the responding CD8^+^ T cells were polyfunctional exhibiting four effector functions simultaneously (Fig. 14b and d).

**TABLE 3:** Participant demographics

Participant	HLA class I		Gender	Age (yr)	Date diagnosed	COVID-19 severity	Time after SARS-CoV-2 recovery (mo)	Highly networked T-cell epitope^*a*^	SARS-CoV-2 protein source^*b*^
	Supertype	Allele(s)							
CVBL06A	HLA-A*02 positive	HLA-A*02:06 HLA-A*24:02	Female	60	26 Mar 2020	NA	1	RTATKAYNV	NC C-terminal domain
CVBL10B	HLA-A*02 positive	HLA-A*02:01 HLA-A*03:01	Male	61	28 Mar 2020	NA	2	IIWVATEGA	NC N-terminal domain
CVBL05A	HLA-A*02 negative	HLA-A*24:02	Female	66	5 Apr 2020	Hospitalized	1	RTATKAYNV	NC C-terminal domain
		HLA-A*24:07						IIWVATEGA	NC N-terminal domain

a Derived peptides tested in *ex vivo* PBMCs.

b For the highly networked T-cell epitope derived peptides.

## DISCUSSION

In order to accelerate the development of a diagnostic assay that can measure T-cell immune responses against global SARS-CoV-2 strains, we identified specific T-cell epitopes which are conserved across circulating viral variants from six global regions. In particular, these epitopes contained amino acid residues that are highly networked indicating their topological importance within the NC and S proteins of the virus (36, 46, 54). By performing an immunoinformatics analysis, we defined 57 T-cell epitopes within the SARS-CoV-2 proteins, of which 11 were unique and non-cross-reactive to seasonal human coronaviruses, that should be considered for detecting a combined SARS-CoV-2-specific CD4/CD8 T-cell immune response (16, 26, 29, 43, 44). Importantly, these highly networked T-cell epitope derived peptides were identified from the regions that lack mutations reported to enhance viral infectivity (30). In addition, we assessed key antigen processing and presentation parameters to further delineate the T-cell epitopes which are most likely to induce an HLA class I-mediated immune response. In conducting this study, we selected the T-cell epitopes restricted to the HLA-A*02:01 and DRB1*07:01 alleles based on their global frequencies. Furthermore, we validated *in vitro* the binding of two HLA-A*02-specific T-cell epitope derived peptides from the highly networked regions of the NC protein to the T2 cell line expressing HLA-A*02:01 molecules. Also, we assessed the HLA class I mediated CD8^+^ T-cell immune response to these peptides by using PBMCs obtained from two SARS-CoV-2 patients 1 to 2 months postrecovery.

The NC and S structural proteins are highly homologous between the viruses from the *Coronaviridae* family due to their importance for viral replication (55–58). A recent study identified a number of T-cell epitopes that are conserved between SARS-CoV-1 and -2 (43). Among the approximately 600 SARS-CoV-2 protein sequences derived from six global regions, we identified highly networked T-cell epitopes that matched more than 95% of the circulating SARS-CoV-2 variants (38–40). This allowed us to select the T-cell epitopes for further analysis with the potential for a universal tool that can detect T-cell responses to worldwide strains of SARS-CoV-2. In addition, these T-cell epitopes are identified from the topologically important sites where molecular interactions between amino acid residues are critical for maintaining the structure and function of the viral proteins; and therefore, these sites are not frequently mutated (36, 59–61). Since these sites are mostly found in the core of the proteins, the highly networked T-cell epitopes selected from these regions are most likely to be protected from proteasomal and lysosomal degradation pathways that shape the T-cell epitope repertoire (62–64).

Currently, a total of 14 mutations have been reported within S protein sequences (30). These mutations define important SARS-CoV-2 clades currently reported in GISAID. Some of these have been predicted to enhance viral infectivity of target cells expressing angiotensin-converting enzyme 2 (ACE2) (30). Most of these mutations are found within subunit 1 of the spike protein where the receptor binding domain (RBD) is located. Also, these mutations define region specific SARS-CoV-2 clades. As our immunoinformatics analysis pipeline selects T-cell epitope derived peptides with high network scores that avoids these mutations, these peptides will detect T-cell immunity regardless of SARS-CoV-2 clade.

By applying our immunoinformatics analysis, we found the T-cell epitopes derived from topologically important regions of the NC (high network scores) correlate with the HLA class I antigen processing and presentation parameters. This indicates the peptides from the NC are likely to induce HLA class I restricted CD8^+^ T-cell response (26, 29, 33). In agreement with this correlation, we observed effector and polyfunctional responses from the CD8^+^ T cells of two SARS-CoV-2 convalescent participants to two peptides from the NC protein. This suggests that our immunoinformatics analysis pipeline identifies immunodominant regions within the SARS-CoV-2 NC protein.

Recent phase I immunogenicity and safety trials of vaccine candidates encoding for the S protein have been shown to induce neutralizing antibodies and IFN-γ T-cell response to SARS-CoV-2 (2–4, 6, 65). However, the polyfunctionality of this T-cell response is unknown. Also, it has been shown that the mRNA vaccine encoding spike-RBD induces primarily CD4^+^ Th1-type response (2). Whether these SARS-CoV-2 vaccines induce polyfunctional CD8^+^ T cells is unclear. This prompted us to identify T-cell epitopes restricted to the HLA class I and II alleles within the S protein of SARS-CoV-2 that can be used to detect polyfunctional T-cell responses. For the S protein, we found 22 T-cell epitope derived peptides most promising for HLA class I restricted immune response despite the lack of correlation between network scores and HLA class I antigen processing and presentation parameters. In addition, the 12-mer peptides derived from the epitopes within the S protein are predicted to bind to HLA class II alleles and could stimulate CD4^+^ T-cell response (29, 33). In particular, six of these 12-mer peptides were sequestered in two B-cell epitopes, suggesting their importance when assessing CD4^+^ T-cell response against SARS-CoV-2. In the future, we will validate the CD4 immune response to these 12-mer peptides using the PBMCs obtained from SARS-CoV-2 convalescent participants.

A recent study by Moderbacher et al. has shown that COVID-19 disease severity is associated with delayed and/or limited SARS-CoV-2-specific CD4^+^ and CD8^+^ T-cell responses during acute infection (66). In contrast, less severe disease is strongly related to a higher proportion of effector CD8^+^ T cells that can produce IFN-γ, an important antiviral cytokine in mucosal sites (66, 67). However, a longitudinal assessment of SARS-CoV-2-specific T-cell immunity during both the acute and the chronic phases of COVID-19 can further delineate the cellular immune response against SARS-CoV-2 and its association with disease severity. In particular, employing the NC and S protein-derived immunogenic peptides that have low homology to seasonal human coronaviruses will allow for the detection of cellular immune responses that are absolutely specific to SARS-CoV-2. Importantly, the highly networked and conserved SARS-CoV-2-specific immunogenic peptides defined from the NC and S protein sequences derived from global viral variants can contribute to this longitudinal assessment of T-cell immunity.

The T-cell epitope derived peptides defined by our immunoinformatics analysis pipeline can also contribute to the development of a “second-generation” vaccine that aims to stimulate combined CD4/CD8 T-cell immune responses (18, 68). The levels of SARS-CoV-2 neutralization antibodies alone do not determine protection against the virus (66). Rather, a coordinated approach that can mount both the virus-specific antibodies and CD4/CD8 immune responses will be effective against SARS-CoV-2 (9, 66, 69, 70). Therefore, the immunogenic peptides selected from the highly networked and conserved T-cell epitopes within the NC and S proteins via our analysis pipeline could be considered as vaccine candidates to elicit CD4/CD8 T-cell immune responses against SARS-CoV-2.

There are several limitations to our study. First, our immunoinformatics analysis pipeline was applied to two specific HLA alleles. However, we identified highly networked T-cell epitopes derived from the NC and S proteins that are predicted to bind to 18 to 24 additional HLA class I and II alleles classified into multiple HLA supertypes/loci. Importantly, these can cover approximately 80 to 100% of the global population, indicating that these peptides can be used internationally for the T-cell immunity detection assays. Second, the protein homology modeling of the NC N-terminal RNA binding domain was based on the SARS-CoV (41). However, this SARS-CoV protein domain was the best predicted template when performing our analysis (41, 71–73). Third, even though we only assessed 12-mer peptides for the HLA class II allele, a previous study has shown that this length can accurately predict the binding to HLA class II molecules (50). Of note, longer peptides which are frequently presented by the HLA class II molecules should include the 12-mer peptides that we identified as being highly networked (51, 74). Lastly, all of the promising T-cell epitope-based peptides identified by our immunoinformatics analysis pipeline require *in vitro* and *ex vivo* assessments to determine whether they induce T-cell responses during and after SARS-CoV-2 infection (9, 15). However, our interim *in vitro* validation showed that our immunoinformatics analysis pipeline has identified two T-cell epitope derived peptides from the NC protein that can stably bind to the HLA-A*02 molecules. Furthermore, the CD8^+^ T cells derived from the SARS-CoV-2 survivors exhibited polyfunctional effector responses to these peptides, which have not been previously identified as promising epitopes for T-cell-mediated immune response by recent studies (16, 26, 29, 43, 44). These *in vitro* and *ex vivo* cellular binding and response studies provide proof of concept that our immunoinformatics analysis pipeline identifies novel T-cell epitopes which can elicit a SARS-CoV-2-specific T-cell response which was polyfunctional. Our future studies will assess T-cell-mediated responses to all of the highly networked T-cell epitope derived peptides identified by our immunoinformatics analysis pipeline. Of note, as the selected peptides are identified within the core of the viral proteins, a subset of them have the potential to be hydrophobic which affects peptide-synthesis. This issue can be overcome by extending the length of the T-cell epitope derived peptides so that they include hydrophilic amino acid residues.

In conclusion, the application of an immunoinformatics analysis pipeline allowed us to identify 57 highly networked T-cell epitopes, of which 11 were unique and non-cross-reactive to seasonal human coronaviruses, from the NC and S proteins which are promising immunogenic peptides for detecting HLA class I- or II-related immune response. Of these peptides, two novel T-cell epitopes from the NC can stably bind to HLA class I molecules and induce polyfunctional effector CD8^+^ T-cell responses. Our findings indicate that our immunoinformatics analysis pipeline can contribute to the development of assays that detect polyfunctional and SARS-CoV-2-specific T-cell responses against diverse SARS-CoV-2 viral strains, distinct from pre-existing seasonal coronavirus immunity. The T-cell immunity assay using our peptides have the potential to detect T-cell immune responses elicited by diverse HLA polymorphisms.

## MATERIALS AND METHODS

### Study approval.

This study was carried out in accordance with the recommendations of the institutional review board at the Western Sydney Department for the Westmead Institute for Medical research (WSLH HREC 2020/ETH0084 and 2020/STE01476). The protocol was approved by this committee. All participants provided written informed consent prior to inclusion in the study.

### Global and regional distribution of HLA class I and II alleles.

An understanding of the worldwide distribution of HLA class I and HLA class II alleles is important when selecting immunodominant epitopes for vaccine candidates against the SARS-CoV-2 global pandemic. Currently, there are more than 18,000 HLA class I and 7,000 class II alleles reported (35). Therefore, we determined the five most prevalent HLA class I and HLA class II alleles worldwide by data curation from The Allele Frequency Net Database (www.allelefrequencies.net) (37). This allowed us to select the dominant HLA-I A*02:01 (HLA-A*02:01) and HLA-II DRB1*07:01 (DRB1*07:01) alleles for inclusion in our immunoinformatics analyses (Fig. 1 and 8). The global frequencies of these alleles are 20 and 12%, respectively. Since the most severely affected regions for SARS-CoV-2 infection are found in Europe and the Americas (75), we applied *in silico* immunoinformatics analysis to identify T-cell epitopes within the NC and S protein sequences which are effective for HLA-A*02:01- and DRB1*07:01-mediated antigen restriction (Fig. 2).

### Genetic variability of circulating SARS-CoV-2.

A universal vaccine targeting different strains of coronavirus is also desirable as new viral strains can emerge from animal-to-human transmission and region-specific genetic diversification. To identify genetically conserved T-cell epitopes for possible vaccine development, we examined the genomic diversity of circulating SARS-CoV-2 isolates from six global regions. Whole-genome sequences (WGS) from NSW, Australia (*n* = 15) (76) were combined with local and global references available from GISAID (https://www.gisaid.org/) (39, 40). As of March 2020, we obtained a total of 607 SARS-CoV-2 genomic sequences from GISAID and aligned them using MAFFT (77). These sequences contained representatives from all major lineages (https://github.com/hCoV-2019/pangolin). The regions encoding the nucleocapsid (NC) and Spike (S) protein sequences were translated and extracted with ambiguous positions removed. The resulting alignments contained 586 and 567 sequences for the NC and S proteins, respectively. These NC and S protein alignments represented data from Asia (*n* = 178), Europe (*n* = 238), North America (*n* = 137), South America (*n* = 10), Africa (*n* = 1), and Oceania (*n* = 39).

A sliding window approach was used to identify all possible 9- and 12-mer peptides derived from the consensus sequences of the N- and C-terminal domains of the NC and the S protein. The percent identity of each amino acid within these 9- and 12-mer peptides was calculated using the Geneious version 8.1.9 (78). From this value the genetic variability of each peptide was then determined (100% identity). The peptides with 0% genetic variability were selected for further immunoinformatics analysis (Fig. 2, 3, and 9). We also compared our T-cell epitope derived peptides to the sequences of SARS-CoV-2 circulating variants listed in the GISAID as of August 2020 (39, 40).

### Protein structure homology modeling of SARS-CoV-2 NC and S proteins.

We modeled three-dimensional structures of the NC and S consensus protein sequences derived from the alignments by using SWIMSS-MODEL (https://swissmodel.expasy.org/) (Fig. 2) (71–73). Using homology-structure modeling, we predicted the N-terminal RNA binding domain of the NC protein structure by using SARS-CoV nucleocapsid template (PDB 1SSK, STML ID 1SSK.1.A) as this was the accurate template for this region (41). For the C-terminal dimerization of the NC protein, the protein structure was modeled by using SARS-CoV-2 coronavirus as a template (PDB 7C22, STML ID 7C22.1.B) (42). We performed automated structure homology-modeling on the S consensus protein sequence by using chain A of SARS-CoV-2 spike protein as the template (S protein: PDB 6VSB.1; STML ID 6VSB.1.A) (73). As the part of this protein modeling, all protein regions were investigated for their genetic identity to SARS coronavirus and other organisms. We only used the protein regions that were related to SARS coronavirus to define T-cell epitopes.

### Identifying highly networked epitopes within the NC and S proteins.

To identify suitable T-cell epitopes as targets for the T-cell immunity assay, we used an immunoinformatics pipeline that combines protein structure-based network analysis and sequence-based HLA class I and II binding prediction within the nonvariable NC and S regions of SAR-CoV-2 (Fig. 2) (36, 46, 47, 54). We used the Network Analysis of Protein Structures (NAPS) program (http://bioinf.iiit.ac.in/NAPS) (79) to quantify structural and spatial importance of each amino acid residue within the tertiary structure models of the N- and C-terminal domains of nucleocapsid and S protein (Fig. 2). We combined networks defined by geometric center (“atom pair contact”) and center of mass (“centroid”) for each amino acid residue within the SARS-CoV-2 protein model (79, 80). The “atom pair contact network” describes physicochemical interactions between an atom-pair within an amino acid residue and the “centroid network” defines the connectivity between center of mass of any two amino acid residues within a protein structure (79, 80). We also calculated the distance from the center of mass of each tertiary protein model to all amino acid residues presented in the protein structure by using CALCOM (http://bioinformatica.isa.cnr.it/CALCOM/input.html) (81–83). This allowed us to quantify the spatial location of each amino acid residue with respect to the center of the tertiary structure of each protein (81–83). We used a total of five parameters derived from NAPS and CALCOM when calculating a network score for each amino acid residue within each protein structure. These parameters are as follows: (i) number of direct neighbors of a geometric center of an amino acid residue (Degree_Atom pair contact network_); (ii) number of direct neighbors of a center of mass of an amino acid residue (Degree_Centroid network_); (iii) a ratio of the degree of interconnectivity: (Betweeness_Centroid network_) calculated by (number of the shortest pathways between a particular amino acid residue and its neighbors)/(the total number of the shortest pathways within the protein centroid network) (Each pathway is weighted based on the distance between two amino acid residues); (iv) a cumulative intermolecular strength of all neighboring amino acid residues connected to a geometric center of a particular amino acid residue within the atom pair contact network (Strength_Atom pair contact network_); and (v) a distance from the center of mass to an amino acid residue within a protein structure (Distance).

By modifying the previously published equations (36), we determined a network score for each 9- and 12-mer peptide derived from the N- and C-terminal domains of the NC and S proteins by using the following equation below: network score for each amino acid residue = (Degree_Atom pair contact network_ + Degree_Centroid network_)/4 + (Betweeness_Centroid network_ + Strength_Atom pair network_)/2 – Distance. All proteins are subjected to proteasomal and lysosomal degradation processes before generating peptide repertoires for further HLA antigen presentation (62–64). To select the peptides which are protected from these degradation pathways, our calculation weighted the position of the amino acids within a protein structure more than the connectivity between amino acid residues. For 9-mer or 12-mer peptides derived from each viral protein, we summed the network scores for each amino acid residue and divided by the corresponding length of the peptides. We then normalized the network scores by subtracting the lowest value. In addition, we calculated the lower 95% confidence interval for mean of the normalized network scores for each peptide length and tertiary protein model. The peptides with a normalized network score above this 95% confidence interval were considered highly networked in this study. The peptides comprised of highly networked amino acid residues within the NC and S proteins were compared across the SAR-CoV-2 isolates available. The 95% confidence intervals for the mean of the normalized network scores for each peptide length and tertiary protein model was computed by using STATA 15.1 (StataCorp, 2017; Stata Statistical Software, release 15 [StataCorp LLC, College Station, TX]) (84). The normalized network scores for each peptide are presented in the main figures and tables.

The consensus sequences of NC-N-terminal, NC-C-terminal and S proteins, and peptide sequences (9- and 12-mers) are available on https://github.com/EunokLee/SARS-CoV-2_data_files_and_acknowledgements. Also, the PDB files from the protein homology modeling can be downloaded from the same github link.

### HLA class I or II binding affinity. (i) Percent bind level.

Peptides representing the immunodominant epitopes with the best network scores were screened *in silico* for their HLA class I or II binding affinity using NetMHCpan-4.0 and NetMHCIIpan-3.2, respectively (Fig. 2) (46, 54). These programs order peptides from the strongest binders to those which do not exhibit any binding capacity to an HLA molecule by predicting their binding affinities (half-maximal inhibitory concentration [IC_50_]) (47). Based on the IC_50_, the algorithm ranks the peptides from the strongest binder to weakest binder. For example, those which are categorized as the top 2 to 10% bind rank are considered binders to HLA class I and II molecules. For the data presentation in this study, we subtracted this percent rank from 100% and presented the resulting value as a percent bind level for each peptide. In other words, the binders to HLA class I and II molecules have a high percent bind level whereas the nonbinders have a low percent bind level. To standardize our selection method for the peptides with predicted binding capacity to HLA class I and II molecules, we calculated the percent bind level that equates to the 95th-percentile threshold for each peptide repertoire. This repertoire consists of 9- or 12-mer peptides derived from the NC N-terminal domain, the NC C-terminal domain, or the S protein. This percentile method allowed us to select the peptides with percent bind levels which are categorized into the top 5% bind level. The 95th percentile was determined by a normal quantile plot (STATA 15.1; StataCorp, 2017) (85).

### (ii) IC_50_ cutoff.

The majority of T-cell epitopes have binding affinities lower than an IC_50_ of 5,000 nM according to the IEDB MHC-I binding predictions (http://tools.iedb.org/mhci/). Therefore, we used the IC_50_ of <5,000 nM as the cutoff, in addition to our 95th-percentile bind level threshold, to determine the peptides with binding potential to HLA class I and II molecules.

### Additional HLA class I-related antigen processing and presentation prediction.

By using IEDB combined predictor (http://tools.iedb.org/processing/), we scored the 9-mer peptides derived from the NC and S proteins that were most likely to be processed for HLA class I mediated antigen presentation (Fig. 2) (86). The IEDB algorithm generates predicted proteasomal cleavage and transporter associated with antigen processing (TAP) scores for individual peptides. High proteasomal cleavage and TAP scores indicate efficient antigen presentation (87). Due to the arbitrary grading for these scores, we derived proteasomal cleavage and TAP scores that equate to the 95th percentile for each 9-mer peptide repertoire derived from the NC N-terminal domain, the NC C-terminal domain, or the S protein. This allowed us to select the peptides with HLA class I-related antigen processing scores which are categorized into the top 5%.

To further delineate T-cell epitopes from the 9-mer peptide repertoire, we predicted HLA class I-mediated antigenicity by using an IEDB analysis tool (http://tools.iedb.org/immunogenicity/) (Fig. 2) (88). This analysis tool scores the immunogenicity by determining the positions and side chain properties of the amino acid residues within a peptide-HLA complex that binds to a T-cell receptor. We used the default setting when performing the immunogenicity prediction. The peptides with high immunogenicity have high prediction scores. Therefore, we used the immunogenicity score that equates to the 95th percentile as the cutoff to identify the peptides with top 5% scores. In addition, we predicted the stability of a complex formed by the peptide and HLA class I molecule (p:HLA) by using NetMHCstabpan-1.0 (89). The predicted stability of p:HLA is reported as the time required for the peptide to dissociate from the HLA class I molecule (p:HLA *t*_1/2_). The percentile cutoffs for HLA class I related antigen processing and immunogenicity were determined by a normal quantile plot (STATA 15.1; StataCorp, 2017) (85).

### Selection of T-cell epitope derived peptides for HLA class I and II immune responses.

The T-cell epitopes selected as promising candidates that can contribute to the development of T-cell immunity assays specific for SARS-CoV-2 had the following parameters: (i) a peptide genetic variability of 0%; (ii) peptide network scores above the threshold (i.e., above lower 95% confidence interval); (iii) a percent bind level to HLA-A*02:01 or DRB1*07:01 above the threshold (i.e., within the top 5%); (iv) IC_50_ of <5,000 nM derived from HLA binding prediction algorithms; and (v) for HLA class I epitopes, at least one score predicted for antigen processing and presentation reaching the top 5%.

### Correlation of the network scores to the HLA class I antigen processing and presentation parameters.

We performed a correlation analysis between network scores and predictions for HLA class I mediated antigen processing and presentation parameters (i.e., proteasomal processing score, TAP score, and HLA-I immunogenicity). The correlation analysis was performed by using STATA 15.1. This analysis was applied to peptide repertoires derived from the N- and C-terminal domains of NC and to the S protein. We investigated the *R*^2^ values to determine the proportion of the peptide repertoire that follows linear regression. Moreover, we determined the association between the network scores and the HLA class I- related antigen processing and presentation parameters by the slopes of the regression.

### Sequence comparison between SARS-CoV-2 T-cell epitope derived peptides and seasonal human coronaviruses.

To determine the homology between T-cell epitope derived peptides (Tables 1 and 2) and four seasonal human coronaviruses (hCoVs; 229E, HKU1, NL63, and OC43), we downloaded all available nucleocapsid and spike glycoprotein sequences of these hCoVs from UniProt database (https://www.uniprot.org/). These hCoV sequences (*n* = 1,353) were compared to the SARS-CoV-2 NC and S protein sequences containing the highly networked T-cell epitope derived peptides by using Geneious version 8.1.9. For the protein sequences derived from each hCoV strain and for each region aligning with the SARS-CoV-2 T-cell epitope derived peptide, a mean percent genetic identity and its 95% confidence intervals were calculated by using STATA 15.1 (78, 85).

### Assessing the binding capacity of the T-cell epitope derived peptides to multiple HLA class I and II alleles and their worldwide population coverage.

As we selected highly networked T-cell epitope derived peptides based on the most prevalent HLA class I and II alleles (HLA-A*02:01 and DRB1*07:01), we assessed whether these peptides can bind to additional HLA class I and II alleles by using NetMHCpan-4.1 (90) and NetMHCIIpan-4.0 (91). For the 9-mer peptides, we predicted the binding affinity to additional HLA-A and HLA-B alleles which are known to cover more than 97% of the global population (92). For the 12-mer peptides, we predicted the binding affinity to additional HLA class II alleles which are known to cover more than 99% of the global population (93). For each of the 9- and 12-mer peptide repertoires derived from the NC and S protein sequences, we derived 95th-percentile threshold of the percent bind level to each of the additional HLA class I and II alleles as described above. This percentile method allowed us to determine the peptides with the top 5% percent bind levels to each of these additional HLA alleles. The HLA class I alleles were categorized into 10 supertypes (45), and the HLA class II alleles were grouped by four loci (51) (Fig. 2 and 6). For the global population coverage, we used the IEDB analysis tool called population coverage (http://tools.iedb.org/population/) (94).

### B-cell epitope prediction.

The consensus SARS-CoV-2 NC and S protein sequences were used to predict B cell epitopes by applying an IEDB analysis tool (http://tools.iedb.org/bcell/). For this analysis, BepiPred-2.0, Sequential B-cell epitope predictor that employs the epitopes determined from crystal protein structures (95). The sequential residues with the scores above the threshold of 0.5 were reported as the B cell epitopes in this study.

### Sequence comparison with other published SARS-CoV-2 T-cell epitopes.

We compared the peptides we detected with high network scores and percent bind level to the HLA class I and II molecules to the T-cell epitopes published by recent studies (16, 26, 29, 43, 44). We aligned these peptide sequences using Geneious version 8.1.9 to identify highly networked peptides which are 100% identical to those recently published (78).

### *In vitro* validation of HLA-binding capacity of T-cell epitope derived peptides. (i) Peptides.

We selected RTATKAYNV and IIWVATEGA from the SARS-CoV-2 NC 9-mer peptide repertoires for *in vitro* validation of HLA-binding capacity. These peptides were selected based on their network analysis, bind level, and HLA class I-mediated antigen processing and presentation parameters. For a positive control, we included an HLA-A*02:01 restricted peptide (NLVPMAVATV) derived from cytomegalovirus (CMV). This positive control peptide was derived from CMV glycoprotein 64, a virion tegument protein that is the main component of the enveloped subviral particle (CMV-pp65). As a negative control, we included an HLA-B*07- restricted peptide, TPRVTGGGAM, selected from the CMV-pp65. All peptides were synthesized from Mimotopes, Australia, at >95% purity. The peptides were suspended in 10% dimethyl sulfoxide (DMSO) and 90% water at a concentration of 10 mM. The suspended peptides were stored at −80°C until use.

### (ii) T2 cell line.

The HLA class I restriction of these peptides was tested by using nonadherent human-derived T lymphoblastoid hybrid cell line (T2; 174 X CEM.T2; ATCC CRL-1992) (52, 53). This cell line is TAP deficient, expressing empty HLA class I A*02 molecules on the cell surface. The cells were cultured in RPMI 1640 (Lonza, BE12-702F) supplemented with 10% fetal bovine serum, referred to as RF10. The binding capacity of the peptides to the HLA-A*02:01 molecules was tested when the T2 cells were in the log phase of growth. The T2 cell line was kindly provided by Rajiv Khanna (QIMR Berghofer Medical Research Institute, Queensland, Australia).

### (iii) Hybridoma.

Mouse BB7.2 (ATCC HB-82) hybridoma cells were used for producing the primary anti-human HLA-A*02 antibody and for the staining of the HLA-A*02 molecules expressed on the T2 cell surface. The hybridoma was maintained and cultured in RF10. For antibody collection, the cells were washed with phosphate-buffered saline (PBS; Lonza, BE17-516F) and resuspended in AIM-V serum free media (Thermo Fisher Scientific, catalog no. 12055091) at 10^6^ cells/ml, followed by an incubation at 37°C for 2 days. The supernatant was collected after the cells were pelleted by centrifugation at 300 × *g* for 5 min and filtered through 0.45-μm syringe filter (Merck Millipore, Darmstadt, Germany). The supernatant was stored at 4°C until use.

### (iv) HLA-peptide binding assay.

The ability of synthetic peptides to stably bind to HLA-A*02:01 molecules on the cellular surface of the T2 cell line was assessed by flow cytometry as previously described (96). Briefly, T2 cells (1 × 10^5^ cells in 100 μl) were incubated for 1 h at 37°C in serum-free AIM-V medium (Thermo Fisher Scientific, 12055091) in the presence of the peptides at the concentrations of 0 μM (no peptide control), 1, 10, and 100 μM. The cells were then incubated for 16 h at 26°C and returned to 37°C for 2 h prior to immunofluorescent staining. The unbound peptides were removed by using cold PBS. The anti-HLA-A*02-specific monoclonal antibody (i.e., BB7.2 supernatant) was added to the T2 cells, followed by incubation at 4°C for 30 min. After being washed three times with cold PBS, the cells were incubated with a goat secondary Alexa Fluor 647-labeled anti-mouse immunoglobulin-specific antibody (Life Technologies, A21236) at 4°C for 30 min. Finally, the cells were washed and resuspended in 200 μl of cold PBS. The geometric mean of fluorescence intensity (gMFI) of the T2 cells were then measured with a BD LSRFortessa flow cytometer (BD Biosciences). In this study, we reported the gMFI relative to a no-peptide control.

### *Ex vivo* evaluation of effector and polyfunctional CD8^+^ T-cell responses to the T-cell epitope derived peptides. (i) Participants and clinical samples.

Previously hospitalized SARS-CoV-2 convalescent patients were recruited from The Westmead Hospital in Westmead, NSW, Australia. For this study, we included two HLA-A*02-positive participants and one HLA-A*02-negative participant (Table 3). At 1 to 2 months after SARS-CoV-2 recovery, peripheral blood samples were collected from these participants in citrate anticoagulant tubes and cryopreserved PBMCs were isolated within 1 h of venipuncture. The PBMCs were isolated by Ficoll density gradient centrifugation. To determine the HLA for each donor, PBMCs were stained using a phycoerythrin-labeled anti-human HLA-A*02 antibody (clone BB7.2; BD Bioscience) for 30 min at 4°C, and the immunofluorescence was measured with a BD LSRFortessa flow cytometer (BD Biosciences). Where possible, the PBMCs derived from the HLA-A*02 negative participant were included as an experimental control.

### (ii) Expansion of peptide-specific T cells.

The PBMCs were thawed in RPMI and then rested overnight in RF10. The rested PBMCs ([3 to 5] × 10^6^ cells) were incubated in the presence of 5 μM of SARS-CoV-2 peptide pools or Epstein-Barr Virus (EBV) peptide mix (MACS GMP PepTivator EBV select, Miltenyi Biotec) for 1 h. Two SARS-CoV-2 peptides derived from the NC (RTATKAYNV and IIWVATEGA; 5 μM for each peptide) were used to stimulate PBMCs for 1 h. The EBV peptide mix (resuspended at 100 μg/ml in DMSO) was used for a positive control (MACS GMP PepTivator EBV Select; Miltenyi Biotec). After the incubation with these peptides, the cells were washed once with RPMI and costimulated using purified anti-human CD28 antibody (1 μg/ml) (clone L293; BD Biosciences). The stimulated cells were cultured in 48-well plates at a density of 2 × 10^6^ cells/ml in RF10 medium supplemented with 100 U/ml IL-2 (Lonza, catalog no. 200-02) for 14 days. The medium was replaced every 72 h with freshly prepared RF10 supplemented with IL-2. The expanded cells were subsequently studied by flow cytometry.

### Detection of effector and polyfunctional CD8^+^ T cells responses to the T-cell epitope derived peptides.

The effector and polyfunctionality of CD8^+^ T cells were evaluated by using the expanded cells that were exposed to the peptides for 14 days. Briefly, the expanded cells were restimulated with individual SARS-CoV-2 peptides or the EBV peptide pool in the presence of costimulatory antibodies (1 μg/ml of anti-CD28 and anti-CD49d; BD Biosciences), monensin (Golgistop, 0.9 μl/ml; BD Biosciences) and brefeldin A (1 μl/ml; BD Biosciences) for 5 h at 37°C. Anti-CD107a/b-FITC antibodies (BD Biosciences) were also added to identify degranulating cells. For the functionality panel, the cells were stained upon stimulation with Live/Dead Fixable Near-IR Dead cell stain kit (Thermo Fisher) and the following conjugated antibodies: anti-CD3-BUV496 and anti-CD8- PerCP-Cy5.5 (BD Biosciences). The cells were then fixed and permeabilized (Cytofix/Cytoperm; BD Biosciences). Subsequently, the fixed cells were stained using anti-IL-2-PerCP-Cy5.5, anti-TNF-α-PE/Cy7, and anti-IFN-γ-PE antibodies (BD Biosciences). The data were analyzed by using FlowJo v10 (Data Analysis Software, LLC). The gating strategy was performed as follows: (i) the lymphocyte population was selected by using FSC-A versus side scatter (SSC) plot; (ii) the single cells were selected in a forward scatter area (FSC-A) versus FSC-height plot; (iii) the dead cells were excluded on the bases of Live/Dead Near-IR fluorescence; and (iv) the CD3^+^ CD8^+^ cells were gated in CD3 versus CD8 dot plots. To study the polyfunctionality of CD8^+^ T cells, CD8 versus CD107a/b, IFN-γ, IL-2, or TNF-α plots were constructed. After the gates for each cytokine profile of CD8^+^ T cells were created, the Boolean gate platform was employed to create all possible cytokine and CD107a/b combinations. For each combination, the resulting data were obtained by subtracting percent cells representing the background in the mock control and CD28/CD49d stimulation. The values below the background were set at 0. For the polyfunctionality analysis, SPICE 6.0 software (https://niaid.github.io/spice/) was used following the technical considerations published by the software developers (97).
